# Effectiveness of cuticular transpiration barriers in a desert plant at controlling water loss at high temperatures

**DOI:** 10.1093/aobpla/plw027

**Published:** 2016-05-06

**Authors:** Ann-Christin Schuster, Markus Burghardt, Ahmed Alfarhan, Amauri Bueno, Rainer Hedrich, Jana Leide, Jacob Thomas, Markus Riederer

**Affiliations:** ^1^Chair of Botany II - Ecophysiology and Vegetation Ecology, University of Würzburg, Julius-von-Sachs-Pl. 3, Würzburg D-97082, Germany; ^2^Department of Botany and Microbiology, College of Science, King Saud University, PO Box 2455, Riyadh 11451, Saudi Arabia; ^3^Chair of Botany I - Plant Physiology and Biophysics, University of Würzburg, Julius-von-Sachs-Pl. 2, Würzburg D-97082, Germany

**Keywords:** Aliphatic compounds, cuticular transpiration, cuticular wax, cutin, desert, minimum conductance, plant cuticle, temperature, transition temperature, triterpenoids

## Abstract

Maintaining the integrity of the cuticular transpiration barrier even at elevated temperatures is of vital importance especially for hot-desert plants. Currently, the temperature dependence of the leaf cuticular water permeability and its relationship with the chemistry of the cuticles are not known for a single desert plant. This study investigates whether (i) the cuticular permeability of a desert plant is lower than that of species from non-desert habitats, (ii) the temperature-dependent increase of permeability is less pronounced than in those species and (iii) whether the susceptibility of the cuticular permeability barrier to high temperatures is related to the amounts or properties of the cutin or the cuticular waxes. We test these questions with *Rhazya stricta* using the minimum leaf water vapour conductance (*g*_min_) as a proxy for cuticular water permeability. *g*_min_ of *R. stricta* (5.41 × 10^−5^ m s^−1^ at 25 °C) is in the upper range of all existing data for woody species from various non-desert habitats. At the same time, in *R. stricta*, the effect of temperature (15–50 °C) on *g*_min_ (2.4-fold) is lower than in all other species (up to 12-fold). *Rhazya stricta* is also special since the temperature dependence of *g*_min_ does not become steeper above a certain transition temperature. For identifying the chemical and physical foundation of this phenomenon, the amounts and the compositions of cuticular waxes and cutin were determined. The leaf cuticular wax (251.4 μg cm^−2^) is mainly composed of pentacyclic triterpenoids (85.2% of total wax) while long-chain aliphatics contribute only 3.4%. In comparison with many other species, the triterpenoid-to-cutin ratio of *R. stricta* (0.63) is high. We propose that the triterpenoids deposited within the cutin matrix restrict the thermal expansion of the polymer and, thus, prevent thermal damage to the highly ordered aliphatic wax barrier even at high temperatures.

## Introduction

The efficient control of water loss to the atmosphere is one of the foremost prerequisites for plant survival and competitiveness in any environment but particularly in deserts. Stomata and cuticle together enable plants to maintain a favourable water status. All above-ground primary parts of terrestrial higher plants are covered by a cuticle. This structure with thicknesses ranging from <1 μm to >20 μm (Jeffree 2006) consists of a matrix of polymeric cutin (cross-linked C_16_ and C_18_ hydroxy fatty and hydroxyepoxy fatty acids) with cuticular waxes embedded within or deposited onto its surface. The typical compounds of plant cuticular waxes are long-chain aliphatics and cyclic molecules, especially pentacyclic triterpenoids (Jetter *et al.* 2006).

The water permeability of the cuticle determines the minimum and inevitable water loss when the stomata are maximally closed at drought stress. Therefore, a low cuticular water permeability is one of the main factors supporting the survival and viability of plants under scarce water supply. Leaf cuticular water permeabilities **[see Supporting Information File 1]** differ widely among species and range from 10^−^^7^ to 10^−^^4^ m s^−^^1^ (Riederer and Schreiber 2001).

The extraction of the cuticular waxes with organic solvents leads to increases in the cuticular water permeability by up to several orders of magnitude (Schönherr 1976; Schönherr and Lendzian 1981), which demonstrates that the waxes constitute the main transport-limiting barrier of cuticles. The thickness of the cuticle or the amount of total cuticular waxes associated with it are not related to cuticular water permeability (Schreiber and Riederer 1996; Riederer and Schreiber 2001). So, the frequent statements in textbooks and in the scientific literature that thicker cuticles or cuticles containing more wax are better barriers against transpiration than thin ones with only small amounts of wax (e.g. Purves *et al.* 2004; Poorter and Garnier 2007; Lüttge 2007; De Micco and Aronne 2012; Smith *et al.* 2012; Jones 2013) are not supported by experimental evidence. The chemical composition of cuticular waxes varies considerably among species. Despite their physiological and ecological importance, the relationship between the composition of cuticular waxes and the water permeability is not yet sufficiently understood (Schönherr 1982; Riederer and Schneider 1990; Kerstiens 2006).

Cuticular permeability is not a fixed entity but is subject to environmental influence. Temperature has a pronounced effect on cuticular water permeability. Particularly in species experiencing high and fluctuating temperatures, the temperature dependence of permeability is a major parameter influencing the physiological and ecological relevance of the cuticle. The effect of temperature on cuticular permeability is significant. In the species studied so far, cuticular permeability increased slightly at temperatures from 15 to ∼35 °C while in the higher temperature range (above ∼35 °C) a drastic increase in the cuticular water permeability was observed (Riederer 2006; Schreiber 2001).

In deserts, high air temperatures, strong irradiation and substantially reduced transpirational cooling due to stomatal closure can cause leaves to reach temperatures up to 50 °C (Smith 1978). As stated by Goodwin and Jenks (2005), it is hard to reconcile the steep increase of cuticular water permeability above the transition temperature observed with many non-desert plant species with the successful adaptation of xerophytes to high-temperature climates. From the knowledge currently available it seems conceivable that at high temperatures the cuticular transport barrier progressively fails and that the survival of the plant is imperilled by excessive cuticular transpiration. This scenario is aggravated by the fact that very often the relative humidity of the air considerably decreases when temperature raises in the course of a day. So, not only a higher permeability of the cuticle but also a higher driving force at elevated temperature lead to a heightened transpirational water loss that may become critical for the plant. However, the impact of temperature on the cuticular water permeability of hot-desert plants has so far not been investigated. Also, there is no study dealing with the cuticular water permeability of a hot-desert plant and its relationship to cuticle chemistry. Consequently, Goodwin and Jenks (2005) argued that studies be needed to elucidate the composition and structure of the cuticles from xerophytic plants and their association with temperature-dependent water permeability.

As a first step to close this gap in knowledge, we investigated the minimum leaf water conductance within the range of ecologically relevant temperatures of the hot-desert plant *Rhazya stricta*. The minimum leaf water conductance at maximally closed stomata is a good proxy for cuticular permeability (Burghardt and Riederer 2003). We also analysed the qualitative and quantitative composition of the cutin and the cuticular waxes of *R. stricta*. This species is an excellent model for this kind of investigations because it is a non-succulent, perennial shrub widespread in the deserts of the Arabian Peninsula and ranging into Iran, Southern Afghanistan and Pakistan (Abd El-Ghani 1997; Deil and Al-Gifri 1998; Emad El-Deen 2005; Al Khamis *et al.* 2012). *Rhazya* *stricta* has recently been identified as a species with a photosynthetic physiology functional without significant limitations under typical desert conditions of daily extremes of heat, light intensity and low humidity (Lawson *et al.* 2013; Yates *et al.* 2014). The field studies of these authors in the native habitat in Saudi Arabia showed a remarkable high-temperature tolerance of *R. stricta* with no decrease in photosynthetic capacity observed up to leaf temperatures of 45 °C. Since equivalent adaptations on the level of the cuticular transpiration barrier may have evolved, this species seems to be a first choice for testing hypotheses on the potential adaptations of cuticular properties to hot-desert growing conditions.

In this work, we look for an answer to the fundamental question whether *R. stricta* as a typical woody hot-desert plant maintains a cuticular transpiration barrier able to control effectively water loss even at very high temperatures. We hypothesize that in *R. stricta* when compared with woody non-desert plants: (i) the cuticle is more efficient as a barrier to water loss, (ii) the cuticle is more resistant to thermal stress and (iii) the amounts and/or composition of cuticular waxes and cutin are peculiar. From these hypotheses, we predict that in comparison with woody non-desert species, in *R. stricta* (i) the minimum leaf conductance at a given temperature is lower, (ii) the temperature-dependent increase of minimum conductance is less pronounced and (iii) the composition of the cuticle is qualitatively and/or quantitatively different. We test Hypothesis (i) by measuring the minimum leaf water conductance at 25 °C and compare it with literature data, Hypothesis (ii) by analysing the effect of temperature on minimum conductance and also compare it with literature data and Hypothesis (iii) by qualitatively and quantitatively determining the composition of the cutin and the cuticular waxes for comparison with that of other species. We also consider the role certain major cuticular wax components of *R. stricta* may have for the physical stability of the cuticle.

## Methods

### Field site and plant material

Leaves of *R.* *stricta* (Apocynaceae) were obtained from original growing sites located at ∼80 km north-east of Riyadh, Saudi Arabia (25°24'35.51”N, 47°14'32.61”E and 25°22'53.80”N, 47°14'20.52”E). The two sampling sites are 3 km apart and at the same altitude. All measured properties did not differ significantly between the two populations. The area lies in the tropical arid climate zone. Macroclimatic data are available from a standard meteorological weather station at Riyadh. The mean annual rainfall (1979–2009) is 88.9 mm (Almazroui *et al.* 2012). The mean annual temperature (1984–2013) is 26.8 °C. The monthly mean of the maximum temperature is 43.7 °C for the warmest month during August (Krishna 2014). The mean annual relative humidity (1980–2000) is 30.4% (Al-Ghobari 2000). Terminal shoots bearing 10–15 fully developed, undamaged leaves from three to five individual plants per site were harvested from November to February (2012–14). The shoots were immediately put into plastic bags and transported to the laboratory within 24 h at room temperature. Afterwards, the samples were stored at 4 °C for up to 3 weeks. *Rhazya* *stricta* leaves are very robust, and neither their visual appearance nor minimum conductance or chemical composition of cuticle components changed with storage time.

Basic leaf morphological traits (mean saturated and dry leaf weights, leaf area shrinkage during leaf dehydration and stomatal densities for the adaxial and abaxial leaf surfaces) of the plant material used in the experiments were determined **[see** **Supporting Information File** **2]**. The leaves were further characterized by the analysis of pressure–volume curves. Values for the relative water deficit (RWD) at the turgor loss point, the osmotic potential at the turgor loss point, the osmotic potential at full saturation, the apoplastic water fraction and the modulus of elasticity were obtained **[see** **Supporting Information File** **3]**.

### Minimum leaf water conductances

The leaf thermal and hydric tolerances were established to delimit the ecophysiologically allowable ranges of temperature and dehydration to be used in experimentation. The maximum quantum yield of photosystem ll in the dark-adapted state was determined as a vitality parameter at different temperatures and dehydration levels. The maximum thermal and hydric tolerances were 50.8 °C and a RWD of 0.78, respectively **[see** **Supporting Information File** **4]**.

The minimum water conductance of whole leaves (*g*_min_) of *R. stricta* was determined. It is defined as the ‘lowest conductance a leaf can reach when stomata are completely closed as a result of desiccation stress’ (Körner 1995). For mechanistic studies looking for relationships between the barrier properties and the chemical and physical characteristics of the cuticle, measuring the water permeability of isolated, astomatous cuticles would be preferable. However, the leaves of *R. stricta* are amphistomatous, and no cuticles without stomatal pores can be obtained. Burghardt and Riederer (2003) compared *g*_min_ measured with whole leaves according to the protocol employed in the present work with the permeabilities of the isolated astomatous leaf cuticles of five woody plant species. In four species (*Acer campestre*, *Fagus sylvatica*, *Quercus petraea* and *Ilex aquifolium*) *g*_min_ and the water permeability of the isolated cuticles were equal. This suggests that the residual stomatal contribution to *g*_min_ was negligible and that the assumption that *g*_min_ can be used as a reliable proxy for cuticular permeability in *R. stricta* is reasonable.

Leaf minimum water conductances were obtained from the consecutive mass loss of desiccating leaves in the dark at low humidity (Burghardt and Riederer 2003). The wounds of cut petioles of saturated leaves were sealed with a high-melting paraffin wax (melting point 68 °C, Fluka, Neu-Ulm, Germany). The sealed leaves were placed in an incubator (IPP 110, Memmert) with controlled temperature. Leaf minimum conductances were determined at 15, 20, 25, 30, 35, 40, 45 and 50 °C for characterizing the effect of temperature on cuticular permeability. The air temperature in the incubator was checked with a digital thermometer (Testoterm 6010, Testo, Lenzkirch, Germany). Silica gel (Applichem, Darmstadt, Germany) was used to control the humidity of the atmosphere in the incubator. The actual fresh weights of the desiccating leaves were determined as a function of time of desiccation using a balance (Sartorius MC-1 AC210S, Sartorius, Göttingen, Germany, precision 0.1 mg). The transpiration rate (or flux density of water vapour, *J* in g m^−^^2^ s^−^^1^) was obtained from the change in fresh weight (ΔFW in g) with time (*t* in s) across the two-sided leaf area (*A* in m^2^):

(1) $$J=\frac{\Delta FW}{\Delta t\;\times \;A}$$

The transpiration rate at each dehydration level was corrected for leaf area shrinkage **[see** **Supporting Information File** **2]**. The water vapour conductance (*g* in m s^−^^1^) was calculated from the transpiration rate (*J*) divided by the driving force of transpiration. The driving force is the difference between the concentration of water vapour (in g m^−^^3^) in the leaf (*c*_wv leaf_) and in the surrounding atmosphere (*c*_wv air_):

(2) $$g=\;\frac{J}{c_{wv\;leaf}-c_{wv\;air}}=\frac{J}{a_{leaf}\;\times \;c_{wv\;sat\;leaf}-a_{air}\times \;c_{wv\;sat\;air}}$$

The water activity of the air (*a*_air,_ dimensionless) and hence the air water vapour concentration (*c*_wv air_) over silica gel are close to zero (Slavík 1974). The water vapour concentration in the leaf is given by the product of the water activity in the leaf (*a*_leaf_) and the water vapour saturation concentration at leaf temperature (*c*_wv sat leaf_). The water activity in the leaf for each dehydration level was deduced from the respective measured water potential (Ψ_leaf_, **[see** **Supporting Information File** **3]**) which can be converted to the corresponding leaf water activity according to:

(3) $$\Psi_{leaf}=\frac{R\;\times \;T}{V_{W}}\;\times \ln a_{leaf}$$

where *R* is the gas constant, *T* is the absolute temperature and *V*_w_ is the molar volume of water (Nobel 2009). Leaf temperature was measured by a leaf temperature sensor with custom-made leaf clip (Walz, Effeltrich, Germany), and the corresponding water vapour saturation concentrations at leaf temperature were derived (Nobel 2009). The boundary layer conductance was determined using a wet filter paper with the same morphology as *R. stricta* leaves. The wet filter paper was assumed to behave like a water surface and, thus, water conductance was solely controlled by the boundary layer resistance. The evaporation rate was determined gravimetrically in analogy to the method used for the leaves. From the evaporation rate, the conductance was derived assuming a maximum driving force between the wet paper and the dry atmosphere (Grace 1989). The boundary layer conductance was 6 × 10^−^^3^ m s^−^^1^ and did not significantly change with temperature. The boundary layer acts as resistance in series, and leaf conductances were corrected accordingly (Slavík 1974).

The conductance of a leaf at a given dehydration state was plotted versus the equivalent RWD **[see** **Supporting Information File** **2]**. At low RWDs, conductances are high and subsequently decline with progressive leaf dehydration until a plateau is reached where they are no longer sensitive to the further decline of RWD. The constant conductance is commonly interpreted as being the result of maximum stomatal closure. It is called the minimum conductance (*g*_min_). The RWD at the stomatal closure point (RWD_SC_) was determined from the leaf drying curves as mean values of the conductances at the breaking point between the declining branch and the plateau.

### Chemical composition of cuticular waxes and cutin

Waxes and cutin were obtained from isolated cuticles (Schönherr and Riederer 1986) and analysed by gas chromatography/mass spectrometry (see Smirnova *et al.* 2013 for details). Leaf discs (area 1 cm^2^) were incubated and vacuum-infiltrated in a solution of pectinase (10 g L^−^^1^, Trenolin Super DF, Erbslöh, Geisenheim, Germany) and cellulase (10 g L^−^^1^, Celluclast, Novozymes, NCBE, University of Reading, UK) in citric acid buffer (0.01 mol L^−^^1^, Applichem, Darmstadt, Germany) adjusted to pH 3. Isolated cuticular membranes were obtained within 2 weeks. After washing in borate buffer (0.01 mol L^−^^1^, Applichem) adjusted to pH 9 and subsequently in deionized water, the cuticular membranes were air-dried and stored for further use.

Cuticular waxes were extracted by immersing the cuticular membranes two times into 5 mL chloroform (≥99.8%, Roth) for 5 min at room temperature. Additional extraction steps did not increase the total wax yield. Tetracosane (≥99.5%, Sigma-Aldrich, Steinheim, Germany) as an internal standard was added to the combined extracts that subsequently were reduced to dryness under a gentle stream of N_2_.

For cutin analysis, the wax-free polymer matrix membranes were depolymerized with a Boron trifluoride–methanol solution (10% BF_3_ in MeOH, Fluka) at 70 °C for 16 h. Dotriacontane (≥98.0%, Sigma-Aldrich) as an internal standard was added before depolymerization. Subsequently, a saturated aqueous solution of NaCl (Applichem) was added, and the resulting mixture was extracted three times with CHCl_3_. The combined CHCl_3_ extracts were dried over anhydrous Na_2_SO_4_ (Applichem), and CHCl_3_ was completely removed under a gentle flow of N_2_.

Wax and cutin samples were derivatized for gas chromatography with *N*,*O*-bis(trimethylsilyl)trifluoroacetamide (BSTFA, Marchery-Nagel, Düren, Germany) in dry pyridine (≥99.5%, Roth) for 30 min at 70 °C. Quantitative analysis was carried out with a gas chromatograph equipped with a flame ionization detector and an on-column injector (7890A, Agilent Technologies, Waldbronn, Germany). Separation of compounds was achieved on a fused-silica capillary column (DB1-ms, 30 m length × 0.32 mm ID, 0.1 µm film, Agilent Technologies) with hydrogen as a carrier gas. The temperature programme for wax analysis was: injection at 50 °C, after 2 min with 40 K min^−^^1^ to 200 °C, after 2 min with 3 K min^−^^1^ to 320 °C and 30 min at 320 °C. The temperature programme for cutin analysis was: injection at 50 °C, after 1 min with 10 K min^−^^1^ to 150 °C, after 2 min with 3 K min^−^^1^ to 320 °C and 30 min at 320 °C. Qualitative analysis was carried out with a gas chromatograph (6890N, Agilent Technologies) equipped with a mass spectrometric detector (5975 iMSD, Agilent Technologies) under the same gas chromatographic conditions except that helium was used as carrier gas.

### Statistical analyses

Statistical analyses were performed using SigmaPlot 13 (Systat Software, San José, CA, USA). The minimum conductances were tested for normal distribution with the Shapiro–Wilk normality test. Data were normally distributed. The statistical parameters (analysis of variance statistics for the regression, regression degree of freedom is 1 for simple linear regressions) for the regression equations were calculated. Pearson product–moment correlation coefficients were determined. Linear polynomial equations ±SE of regression (leaf shrinkage, pressure–volume analysis) were used to correct the minimum conductance.

## Results

### Leaf drying curves and minimum conductances

**Figure 1.**
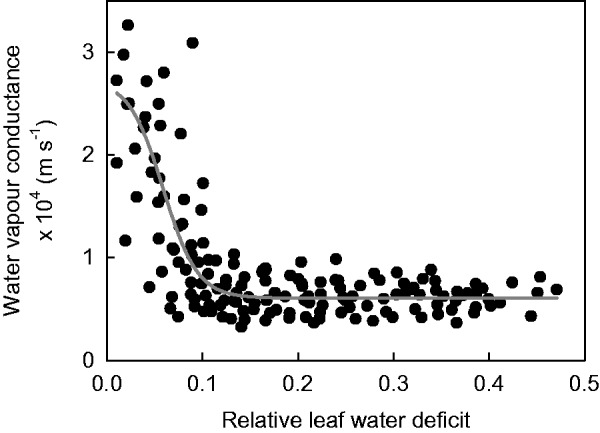
Leaf conductance to water vapour as a function of the relative water deficit (RWD). Each point represents an individual measurement obtained from leaf drying curves at 30 °C with a total of 12 leaves. An exponential four-parameter sigmoid curve is fitted merely to guide the eye. The RWD at maximum stomatal closure is marked by the transition between the declining phase and the plateau phase of leaf conductances. After maximum stomatal closure, leaf conductances remained at a minimum and were constant.

Detached leaves of *R. stricta* were exposed to dry air, and the water loss was measured as a function of dehydration time. For each pair of consecutive data points along the leaf drying curve, transpiration (water loss) rates were calculated according to Equation 1. The respective minimum water vapour conductance was derived from the individual transpiration rates (Equation 2). High conductances characterized the initial phase of the leaf drying curves. Subsequently, with progressing leaf dehydration, conductances declined until a plateau was reached where they were no longer sensitive to a further decline of RWD (Fig. 1). This constant and low conductance is termed the minimum conductance (*g*_min_) at maximum stomatal closure.

High temperatures are a predominant stress factor in the hot and dry natural habitat of *R. stricta*. For this purpose, leaf drying experiments were performed in the range of air temperatures from 15 to 50 °C. For calculating conductances, the actual leaf temperature at a given air temperature was used because it is this value that determines the driving force for transpiration during the phase of maximum stomatal closure. Leaf temperatures were only slightly lower than the corresponding air temperatures at 15° and 20 °C. Increasing air temperatures raised the leaf-air temperature differences (Δ*T*_leaf-air_ in K) that finally reached a value of −1.36 K at an air temperature of 50 °C (Table 1).

**Table 1.** Leaf to air temperature difference during the phase of maximum stomatal closure as a result of desiccation stress, minimum conductances (*g*_min_) and RWDs at maximum stomatal closure (RWD_SC_) obtained from leaf drying curves depend on air temperature (*T*_air_). Each value represents the mean ± SD (*n* ≥ 17). Minimum conductances are expressed in velocity and molar units, a conversion table can be found in Pearcy *et al.* (1989).

*T*_air_ (°C)	Δ*T* (K)	*g*_min_ × 10^5^ (m s^−1^)	*g*_min_ × 10^3^ (mol m^−2^ s^−1^)	RWD_SC_
15	−0.13 (±0.20)	4.27 (±1.03)	1.81	0.12 (±0.03)
20	−0.11 (±0.19)	5.08 (±1.15)	2.12	0.12 (±0.03)
25	−0.29 (±0.23)	5.41 (±1.36)	2.22	0.15 (±0.04)
30	−0.37 (±0.24)	5.96 (±1.30)	2.40	0.14 (±0.04)
35	−0.42 (±0.24)	6.87 (±1.86)	2.73	0.18 (±0.04)
40	−0.64 (±0.34)	7.74 (±2.21)	3.02	0.19 (±0.04)
45	−0.94 (±0.37)	8.72 (±2.09)	3.35	0.25 (±0.06)
50	−1.36 (±0.42)	10.30 (±2.48)	3.88	0.45 (±0.09)

**Figure 2.**
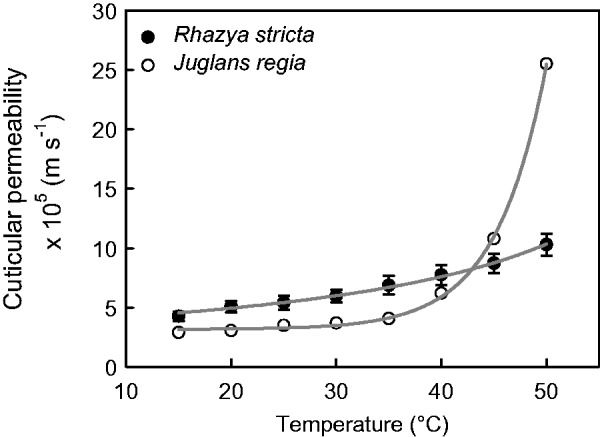
Minimum conductances (*g*_min_) from *Rhazya stricta* leaves obtained from leaf drying curves as a function of air temperature showed a continuous change of cuticular permeability over the whole temperature range from 15 to 50 °C. The cuticular permeance obtained from isolated cuticular membranes from *Juglans regia* leaves (Burghardt and Riederer 2006), a characteristic representative of deciduous trees from a temperate climate, increases steeply above 35 °C.

The RWD at which stomata were maximally closed (RWD_SC_) was derived from the transition between the declining phase and the plateau phase of the curve (Fig. 1). In the temperature range from 15° to 40 °C, stomata closed maximally at RWDs between 0.1 and 0.2. At higher temperatures, maximum stomatal closure shifted to higher RWDs up to 0.45 at 50 °C (Table 1). The minimum conductance at 25 °C was 5.41 (±1.36) × 10^−^^5^ m s^−^^1^ (*n* = 17). Minimum conductances increased with air temperatures by a factor of 2.4 between 15 and 50 °C from 4.27 × 10^−^^5^ m s^−^^1^ to 1.03 × 10^−^^4^ m s^−^^1^ (Table 1). For comparison, also, the corresponding minimum conductances in molar units are given in Table 1. The cuticular permeability of *R. stricta* continuously increased with temperature without an abrupt change in slope (Fig. 2).

### Chemical composition of cuticular waxes and cutin

Plant cuticles consist of a polymer matrix (cutin) and associated solvent–soluble lipids (cuticular waxes). The waxes and the cutin of the *R. stricta* leaf cuticles were analysed qualitatively and quantitatively to detect potential relationships between cuticular transpiration and the composition of the cuticle*.* The total leaf cuticular wax coverage (Table 2) was 251.41 (±38.97) µg cm^−^^2^ (mean ± SD, *n = *5). The cuticular wax was mainly composed of pentacyclic triterpenoids (85.2% of the total wax) with ursolic acid (40.8% of total wax, 48.0% of triterpenoids), echinocystic acid (20.9 and 24.5%) and oleanolic acid (19.1 and 22.4%) as the primary constituents. Only a minor fraction of the wax contained long-chain aliphatic compounds with chain lengths ranging from C_20_ to C_33_ (3.4% of the total wax). Within this fraction, alkanes were the main compound class (2.5% of the total wax, 73.6% of the aliphatics), and hentriacontane was the major constituent (0.9 and 27.8%) of the aliphatic fraction (Table 2). The weighted average chain-length of the very-long-chain aliphatic wax components was 28.5.

**Table 2.** The leaf cuticular waxes of *Rhazya stricta* are composed of a relatively small aliphatic (3.4% of total wax) and a very large triterpenoid (85.2%) fraction. Each value represents the mean ± SD (*n* = 5 biological replicates).

Compound class	Chain length	Coverage (μg cm^−2^)
Alkanes		
	23	0.36 ± 0.11
	25	0.30 ± 0.20
	26	0.29 ± 0.17
	27	0.20 ± 0.12
	28	0.13 ± 0.07
	29	0.28 ± 0.11
	30	0.22 ± 0.04
	31	2.38 ± 0.75
	32	0.37 ± 0.10
	33	1.76 ± 0.72
Total		6.30 ± 2.15
Primary alcohols		
	20	0.64 ± 0.43
	22	0.42 ± 0.31
	24	0.07 ± 0.02
	28	0.14 ± 0.03
	29	0.10 ± 0.01
	30	0.40 ± 0.10
Total		1.78 ± 0.78
Fatty acids		
	20	0.23 ± 0.04
	22	0.14 ± 0.05
	24	0.11 ± 0.05
Total		0.48 ± 0.12
Total aliphatics		8.56 ± 2.93
Triterpenoids		
Taraxerol		0.95 ± 0.19
α-Amyrin		0.27 ± 0.07
Erythrodiol		2.60 ± 1.26
Uvaol		2.27 ± 0.32
Oleanolic acid		48.05 ± 6.18
Betulinic acid		2.47 ± 1.09
Ursolic acid		102.69 ± 12.73
Echinocystic acid		52.56 ± 12.57
Hederagenin		2.26 ± 0.60
Total triterpenoids		214.12 ± 31.20
Not identified		28.72 ± 6.34
*Total wax*		251.41 ± 38.97

Total coverage of cutin monomers was 340.60 (±33.14) µg cm^−^^2^ (*n = *5). Various ω-hydroxy fatty acids with a mid-chain hydroxyl group were predominant (57.1% of the total cutin) followed by ω-hydroxy fatty acids with a mid-chain epoxy group (23.0%). 9/10,16-Dihydroxyhexadecanoic acid (49.7% of the total cutin) and 9,10-Epoxy-18-hydroxyoctadecanoic acid (22.3%) were the major constituents. C_16_ monomers were predominant over C_18_ monomers at a ratio of 1.7 (Table 3).

**Table 3.** The leaves of *Rhazya stricta* are covered by 340.60 μg cm^−^^2^ of total cutin polymer that belongs to the widespread C_16_/C_18_ type. Each value represents the mean ± SD (*n* = 5 biological replicates).

Cutin monomers	Coverage (μg cm^−2^)
Fatty acids	
Hexadecanoic acid (C_16_)	1.13 ± 0.30
Octadecanoic acid (C_18_)	0.79 ± 1.09
Octadec-9-enoic acid (C_18:1_)	0.71 ± 0.32
Nonadec-10-enoic acid (C_19:1_)	0.63 ± 0.24
Eicosanoic acid (C_20_)	Traces
Docosanoic acid (C_22_)	0.33 ± 0.31
ω-Hydroxy fatty acids	
16-Hydroxyhexadecanoic acid (C_16_)	1.71 ± 0.22
16-Hydroxyhexadec-9-enoic acid (C_16:1_)	0.61 ± 0.27
18-Hydroxyoctadeca-9,12-dienoic acid (C_16:2_)	2.77 ± 0.18
ω-Hydroxy fatty acids with midchain hydroxyl group	
9 or 10,15-Dihydroxypentadecanoic acid (C_15_)	2.34 ± 0.15
9 or 10,16-Dihydroxyhexadecanoic acid (C_16_)	169.11 ± 22.95
9 or 10,17-Dihydroxyheptadecanoic acid (C_17_)	1.03 ± 0.24
9 or 10,18-Dihydroxyoctadecanoic acid (C_18_)	7.19 ± 0.89
9,10,18-Trihydroxyoctadecanoic acid (C_18_)	14.77 ± 7.52
9,10,18-Trihydroxyoctadec-12-enoic acid (C_18:1_)	0.12 ± 0.20
9 or 10,19-Dihydroxynonadecanoic acid (C_19_)	traces
ω-Hydroxy fatty acids with midchain epoxy group	
9,10-Epoxy-18-hydroxyoctadecanoic acid (C_18_)	75.88 ± 10.02
9,10-Epoxy-18-hydroxyoctadec-12-enoic acid (C_18:1_)	2.46 ± 0.56
ω-Hydroxy fatty acids with midchain oxo group	
16-Hydroxy-9/10-oxo-hexadecanoic acid (C_18_)	6.19 ± 2.15
α,ω-Dicarboxylic acids	
Hexadecane-1,16-dioic acid (C_16_)	0.76 ± 0.22
Octadecane-1,18-dioic acid (C_18_)	Traces
α,ω-Dicarboxylic acids with midchain hydroxyl group	
7(8)-Hydroxyhexadecane-1,16-dioic acid (C_16_)	4.37 ± 2.21
7(8)-Hydroxyoctadecane-1,18-dioic acid (C_18_)	2.76 ± 0.42
7(8),9(10)-Dihydroxyoctadec-12-ene-1,18-dioic acid (C_18:1_)	0.51 ± 0.07
Phenolics	
Coumaric acid	15.61 ±1.35
Coumaric acid derivative	14.09 ±3.35
Glycerol	0.23 ± 0.03
Unidentified compounds	14.49 ± 2.97

## Discussion

In the ecological literature and textbooks, it is often claimed that desert plants are specifically adapted to reduce transpiration by a combination of a particularly efficient stomatal control and the presence of a cuticular transpiration barrier with extra-low permeability. Intuitively, this seems to be plausible, but it has never been tested whether the cuticular transpiration barrier of desert plants is indeed more efficient than that of species growing in more humid habitats. The results obtained in the present study with *R. stricta* growing in very hot and arid regions allow testing this assumption.

### Is the cuticle of *R.* *stricta* specifically efficient as a barrier to water loss?

**Figure 3.**
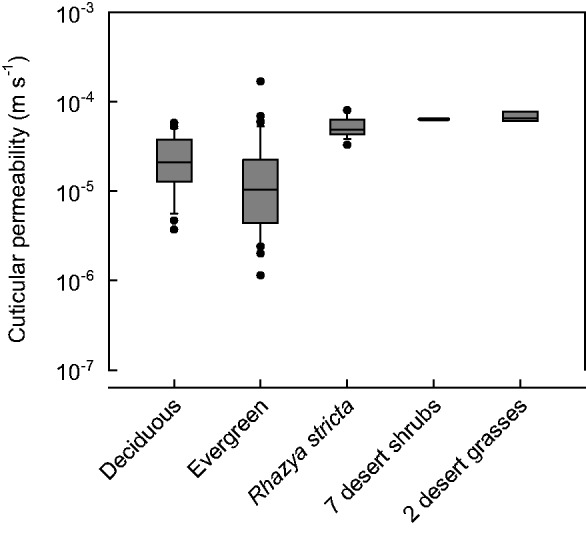
Comparison of literature data of cuticular permeabilities and/or minimum conductances with the minimum conductance obtained from *Rhazya stricta* leaves. The mean values of 12 deciduous woody plant species (21 data points) and the mean values of 13 evergreen woody plant species (44 data points) are compared with 17 single measurements conducted with *R. stricta* leaves. For desert plants, the mean minimum conductances of two desert grass species (three data points) and an average value for seven unspecified evergreen desert shrubs, are available for comparison. For individual values [**see Supporting Information File 5**].

To answer this question, we measured the cuticular transpiration of *R. stricta* leaves under strictly controlled conditions. At 25 °C, the minimum leaf conductance to water vapour is 5.41 (±1.36) × 10^−^^5^ m s^−^^1^ (*n* = 17). This means that it is not specifically efficient as a barrier to water loss because it lies within the upper range of cuticular permeabilities and *g*_min_ measured so far for a large set of woody species from various habitats (Fig. 3, **[see** **Supporting Information File** **5]**). The median of cuticular permeabilities of 12 deciduous and 13 evergreen woody species is 2.10 × 10^−^^5^ and 1.05 × 10^−^^5^ m s^−^^1^, respectively, which is lower than the mean *g*_min_ for *R. stricta*. The upper quartiles of the data used for comparison and the variability of the *R. stricta* data partially overlap. It is difficult to assess this finding because only scarce information on *g*_min_ of desert plants is available. Körner (1994, 1995) reported an average value of 6.4 × 10^−^^5^ m s^−^^1^ for seven unspecified evergreen desert shrub species. Minimum conductances were also published for the desert grasses *Digitaria californica* (6.09 × 10^−^^5^ and 7.72 × 10^−^^5^ m s^−^^1^) and *Eragrostis lehmanniana* (6.51 × 10^−^^5^ m s^−^^1^, Smith *et al.* 2006). The *g*_min_ of *R. stricta* agrees with this very limited pool of data for desert species. From the distribution of the data **[see** **Supporting Information File** **5]** the probability can be estimated that for not yet studied woody plants a cuticular permeability lower than that of *R. stricta* will be found in the future. This probability is 0.93. So, the prediction that the minimum conductance of *R. stricta* leaves is lower than that of non-desert plants and the common notion that desert species should have an exceptionally effective cuticular transpiration barrier are not corroborated by our data.

### Is the cuticle of *R.* *stricta* more resistant to thermal stress than that of other species?

Very high air temperatures are characteristic of the climate of hot deserts and represent a pronounced stressor for all organisms living there. Organisms whose barrier against transpirational water loss consists of waxes embedded within a polymer matrix (plants and arthropods; Hadley 1972, 1981, 1989) are prone to adverse temperature effects. The waxes undergo a solid to liquid transition in the range from 70 to 85 °C in plants (Merk *et al.* 1998) and, for instance, from 35 to 40 °C in the desert *Drosophila mojavensis* (Gibbs *et al.* 1998). These transitions may compromise the functioning of the waxy transpiration barrier. In the past, some work has been devoted to answering the question to what degree the cuticular transpiration barrier of plants is critically affected by high temperatures. However, in contrast to its ecological and physiological relevance, no work on this topic has so far been done with desert plants.

**Figure 4.**
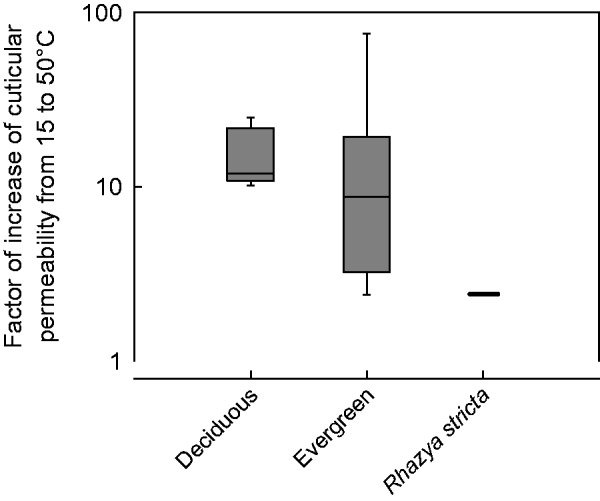
Comparison of literature data of the factor of increase of the cuticular permeabilities from 15 to 50 °C with the increase factor obtained for *Rhazya stricta* leaves. The factors of five deciduous woody plant species and nine evergreen woody plant species are compared with the factor obtained with *R. stricta* leaves.

The present work shows that the temperature dependence of the leaf *g*_min_ of the hot-desert plant *R. stricta* is strikingly different from that of other woody plants from temperate habitats studied so far. In comparison with *Juglans regia* (Burghardt and Riederer 2006), a characteristic representative of deciduous trees from a temperate climate, the change in cuticular permeability is continuous over the whole temperature range from 15 to 50 °C while the permeability of the latter species increases steeply above 35 °C (Fig. 2). This difference becomes even more apparent when the factors are compared by which the cuticular permeabilities of *R. stricta* and non-desert woody plants increase from 15 to 50 °C (Fig. 4). While for *R. stricta* this factor is 2.4, the cuticular permeabilities of deciduous and evergreen non-desert plants (data from Riederer 2006) increase by factors of 12 and 9 (medians), respectively. From the distribution of the values from non-desert plants, the probability can be estimated that for not yet studied woody plants factors higher than that of *R. stricta* will be found in the future. This probability is 0.86. The results from the temperature-dependent measurements of cuticular permeability corroborate the hypothesis that the *R. stricta* leaf cuticle is more resistant to thermal stress than that of woody species from non-desert habitats.

Our data cannot only be used to describe temperature effects on permeability, but they also allow a deeper analysis of the nature of the thermal effects on the properties of the transpiration barrier. Like all processes involving the diffusion of molecules in any medium (solid, liquid or gaseous), cuticular water permeability inevitably increases with temperature. For instance, the diffusion coefficient of water molecules in water increase from 1.77 × 10^−^^9^ m^2^ s^−^^1^ at 15 °C to 3.96 × 10^−^^9^ m^2^ s^−^^1^ at 50 °C (Holz *et al.* 2000). The velocity of diffusion in a liquid is mainly determined by the viscosity of the medium and, therefore, increases when the viscosity decreases at higher temperatures. For plant cuticles, this relationship does not apply. Here, the diffusion of water takes place in an organic solid (cutin, waxes). The mechanisms governing diffusion process in this case are fundamentally different from that in a liquid. In organic solids, the concept of non-Stokesian diffusion applies. This theory states that the mobility of water molecules in a solid medium, *e.g.* a polymer, the lipid parts of biomembranes or waxes depends on the availability of free volumes, i.e. ‘holes’ in between the hydrocarbon chains (Stein 1986). During their thermal motion water molecules jump from one of these holes to another and, thus, proceed in the medium. Diffusion will be high if there are many holes if they appear and disappear at high rates and if the probability that a hole large enough to accommodate the diffusing water molecule appears is also high (Stein 1986). The appearance and disappearance of free volumes within an aliphatic solid is due to the thermal motion of its hydrocarbon chains. At higher temperature, the hydrocarbon chains move faster (especially laterally), open up more free volumes at a higher frequency and the holes appearing become larger. This is the mechanism by which temperature accelerates the diffusion of water across solid organic materials including the plant cuticle. Consequently, temperature effects on cuticular permeability can be analysed based on this concept.

The velocity of a water molecule crossing the cuticle and, as a result, cuticular permeability depends on the rate by which the molecule jumps from one free volume to another. This means that the temperature-dependence of cuticular permeability can be analysed by the Arrhenius formalism which relates rate constants to temperature. A plot of the natural logarithm of the rate constant (in this case cuticular permeability) *vs.* the reciprocal of the absolute temperature generally yields a straight line. Arrhenius plots are linear as long as the diffusion mechanism remains the same irrespective of changing temperature. A sharp deviation from linearity with a marked shift in the slope of the Arrhenius plot indicates a change in the diffusion mechanism mostly due to a change in the properties of the surroundings of the diffusing water molecule.

**Figure 5.**
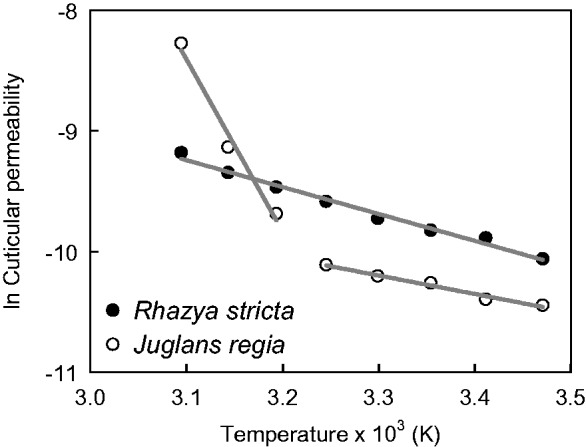
Arrhenius graphs created by plotting the natural logarithm of cuticular permeabilities versus the inverse absolute temperature. Each point represents for a given temperature the mean minimum conductance of *Rhazya stricta* leaves (ln *g*_min_ = –2.332 (±0.338) – (2229.163 (±103.223) × 1/*T*), ±SE of regression, *F* = 466.370, *P* < 0.001, *r*^2 ^=^ ^0.987, *n* = 8) and the mean cuticular permeance of *Juglans regia* leaves (Burghardt and Riederer 2006).

The present work’s results show that the minimum conductance of *R. stricta* steadily increases with temperature from 15 to 50 °C (Fig. 2). In contrast to this, the cuticular permeability of *J. regia* leaves raises only slightly between 15 and 35 °C and at higher temperatures quite steeply (Fig. 2). When the data from both species are plotted according to the Arrhenius formalism, the difference between the two types of temperature dependence becomes even more evident (Fig. 5). While the plot for *R. stricta* is linear over the whole temperature range, the plot for *J. regia* is divided into two branches one ranging from 15 to 35 °C and the second from 40 to 50 °C. Such biphasic Arrhenius plots have also been obtained for all the woody species used for comparison (Fig. 4). So, it can be concluded that in contrast to non-desert woody species, the diffusion of water across the *R. stricta* cuticle is controlled over the whole range of ecophysiologically relevant temperatures by a barrier which does not abruptly change its physical properties at a given temperature. This further supports the hypothesis that the cuticle of *R. stricta* is more resistant to thermal stress than that of other comparable species.

Is it possible to deduce from this finding insights into what makes *R. stricta* cuticles different from others regarding thermal stability? Several hypotheses have been put forward to explain the steep increase of cuticular permeability at temperatures above 35 °C and the concomitant sharp upward bend of the Arrhenius plot observed in all species investigated so far. Eckl and Gruler (1980) attributed it to phase changes of the cuticular waxes. It was also argued that the steep increase of water permeabilities at higher temperatures be due to the swelling of the polysaccharide fraction of the cuticular matrix opening up new pathways for the diffusion of water from the outer epidermal cell wall to the surrounding atmosphere (Riederer 2006). Finally, another hypothesis proposes that the transition is caused by divergent extents of the thermal volume expansion of the cuticular matrix and the associated waxes stressing the wax deposited in the cutin and thus leading to imperfections of the waxy transpiration barrier (Schreiber and Schönherr 1990). The latter hypothesis postulates that mechanical forces act within the cuticle which, at a given transition temperature, open up new pathways for the diffusion of water from the leaf interior to the atmosphere. So in *R. stricta*, the lack of a sudden much steeper increase of water permeability at higher temperatures may be due to a higher mechanical stability of its cuticle. This proposal can be discussed in the context of the chemical composition of the cuticle and its effect on its internal structure and mechanical properties.

### Is the composition of the cuticle of *R.* *stricta* qualitatively and/or quantitatively different from that of other species?

One of the starting hypotheses of this study was that the amounts and/or composition of cuticular waxes and cutin of *R. stricta* are peculiar in adaptation to its hot and arid habitat. The chemical analysis of the cutin polymer of *R. stricta* leaves does not support that hypothesis as it reveals a typical profile of cutin monomers dominated by C_16_ and C_18_ ω-hydroxy fatty acids substituted with mid-chain hydroxyl and epoxy, and end-chain hydroxyl groups. This mixed C_16_- and C_18_-type cutin with 9/10,16-Dihydroxyhexadecanoic acid and 9,10-Epoxy-18-hydroxyoctadecanoic acid as primary monomers (Table 3) is widespread in the leaf cuticles of many plant species irrespective of their habitats (Holloway 1982, 1984; Pollard *et al.* 2008). So, the cutin composition of *R. stricta* cannot be interpreted as being specific for this hot-desert plant.

The cuticular wax composition of *R. stricta* is qualitatively also quite common. The aliphatic wax fraction consists of long-chain *n*-alkanes, primary alkanols and alkanoic acids that occur in the majority of plant species (Baker 1982; Jetter *et al.* 2006). Also, the chain-lengths and chain-length distributions of the aliphatic compound classes are not unusual (Jetter *et al.* 2006). The notion that desert plants tend to have longer average chain-lengths than plants from cooler climates (Bush and McInerney 2015) is not supported by the data from *R. stricta*. The qualitative composition of the cyclic fraction of the wax is not peculiar. The cyclic constituents of the *R. stricta* leaf cuticular wax are taraxerol, α-amyrin, erythrodiol, uvaol, oleanolic acid, betulinic acid, ursolic acid, echinocystic acid and hederagenin, which are quite common in many other plant species (Buschhaus and Jetter 2011; Szakiel *et al.* 2012).

In contrast to the qualitative the quantitative composition of the cuticular wax of *R. stricta* is uncommon although not singular. The wax belongs to a less common type consisting to an extremely high proportion of pentacyclic triterpenoids. In *R. stricta*, triterpenoids make up 85.2% of the total wax. In contrast, the aliphatic fraction is minor and contributes a low fraction of only 3.4% to the total wax (Table 2). This quantitative composition sets *R. stricta* apart from many woody species investigated so far where the leaf cuticular wax is mainly composed of aliphatic components (Merk *et al.* 1998; Wissemann *et al.* 2007; Dragota and Riederer 2008; Jetter and Riederer 2016).

The knowledge of potential functions of triterpenoids has deepened over the last years. Experiments with the fruits of tomato wax mutants (Vogg *et al.* 2004; Leide *et al.* 2007, 2011), *Arabidopsis thaliana* mutant leaves (Buschhaus and Jetter 2012) and eight evergreen woody species (Jetter and Riederer 2016) suggest that the contribution of triterpenoids to the efficacy of the transpiration barrier is small or absent. These findings agree with a model for the molecular structure of cuticular waxes proposed earlier (Riederer and Schneider 1990; Riederer and Schreiber 1995). According to this model, cuticular waxes are multiphase systems with highly ordered crystalline zones and amorphous zones in between. The crystalline zones of the wax are thought to consist of long-chain aliphatic molecules while chain ends, short-chain aliphatics and cyclic compounds should accumulate in the amorphous zone located in between the crystallites. Casado and Heredia (1999) and Tsubaki *et al*. (2013) argued that triterpenoids have a low-molecular order and consequently can be expected to pertain to the amorphous zone. This model also predicts that it is mainly the aliphatic fraction of the cuticular waxes, which represents the cuticular transpiration barrier that was confirmed by Jetter and Riederer (2016). When this model was established no species were known with cuticular waxes dominated to a very high degree by triterpenoids. So, the structural model for the cuticular wax has to be extended for taking into account very high-relative contents of triterpenoids. In *R. stricta*, triterpenoids are 25 times more abundant than the aliphatic compounds and, thus, this material must be predominantly present in largely pure deposits and not mixed with the aliphatic portion of the wax.

So, the question is what functions these extensive triterpenoid deposits may have in the cuticle. Triterpenoids are nearly exclusively located within the cutin polymer matrix (Jetter *et al.* 2000; Buschhaus *et al.* 2007; Jetter and Riederer 2016). Exceptions are species whose leaves or stems are covered by extensive deposits of epicuticular triterpenoid crystals (Markstädter *et al.* 2000). Recently, Tsubaki *et al.* (2013) studied the fruit of *Diospyros kaki* where the triterpenoids are also mainly deposited in the interior of the cuticle. They proposed that triterpenoids and cutin be highly compatible with each other. So, triterpenoids and cutin intimately intermix, and the triterpenoids fill voids between the cutin polymer strands. This increases the density and mechanical strength of the cuticular matrix as a whole. Similar suggestions have been made for the functioning of the triterpenoid oleanolic acid in the grape berry cuticle (Casado and Heredia 1999) and of flavonoids in tomato fruit cuticles (España *et al.* 2014).

**Figure 6.**
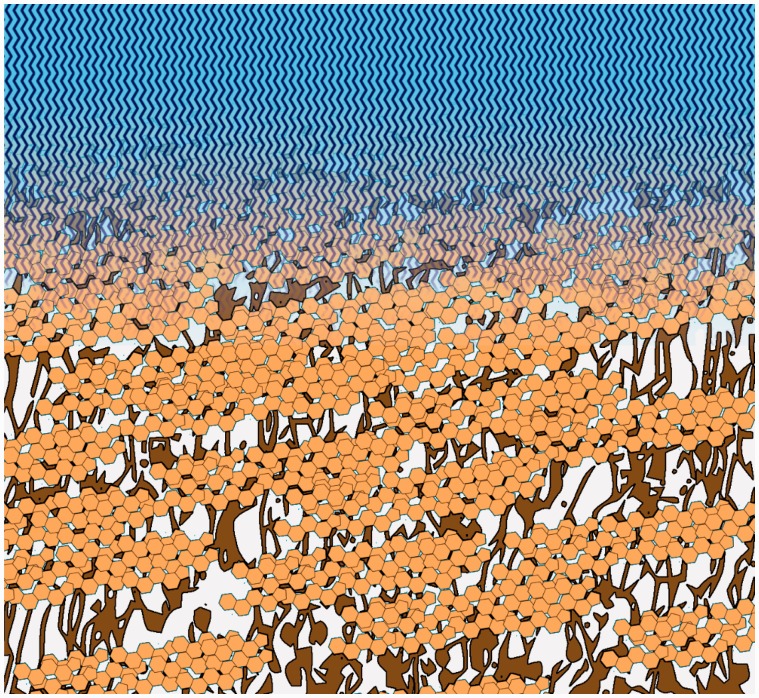
Schematic model depicting the localization and functioning of triterpenoid fillers in the cuticular matrix. Triterpenoids (orange) form extended deposits within the cutin polymer (brown) which fixate the polymer strands. This mechanical enhancement reduces the thermal stress on the layer of aliphatic cuticular waxes (blue) thus maintaining its barrier properties even at elevated temperature.

Frequently, dense materials of organic or inorganic origin, called fillers, are added to amorphous technical polymers for enhancing their strength under mechanical or thermal stress (Xanthos 2010; McCrum *et. al.* 1997). These fillers occupy partially voids between the polymer strands and thereby fixate the latter’s location within the polymer network during stress. Triterpenoids can be considered as perfect fillers for a biological amorphous polymer like cutin. Their melting points are high (e.g. 285–288 °C for ursolic acid, Windholz *et al.* 1983) and their structural arrangement remains unaffected by temperatures up to 100 °C (Casado and Heredia 1999). Consequently, triterpenoid deposits are solid over the whole range of temperatures even a desert plant like *R. stricta* may experience. The mass-based triterpenoid-to-cutin ratio of *R. stricta* is 0.63 which means that 63% of the cutin polymer is associated with agglomerations of triterpenoids that fix the position of the cutin chains and, thus, reinforce the cuticular matrix over the whole range of physiological temperatures (Fig. 6).

So, we formulate the hypothesis that the intracuticular triterpenoids counteract as fillers the thermal expansion of the cutin polymer which, in the absence of reinforcement, might mechanically stress the waxes embedded within the cutin. This stress may introduce imperfections into the waxy transpiration barrier and, thereby, open up new pathways for the diffusion of water molecules to the atmosphere. This assumption is supported by the work of Schreiber and Schönherr (1990) who studied the thermal expansion of the isolated cuticles of 12 plant species. They found that at higher temperatures the ‘volume expansion of the polymer matrix exceeded that of the waxes because the moderately increasing expansion coefficients of the waxes could not compensate for the sudden increase of the expansion coefficient of the polymer matrix’ and concluded that the difference between the two volume expansion coefficients may lead to defects (Schreiber and Schönherr 1990).

In this context, it is intriguing that in contrast to the other species studied so far the minimum leaf conductance of *R. stricta* does not abruptly increase above a certain transition temperature (Fig. 2). The functionality of the cuticular transpiration barrier of *R. stricta* is preserved even at elevated temperatures. Furthermore, the linearity of the Arrhenius plot (Fig. 5) indicates that in contrast to all other species the diffusion mechanism and/or the environment are identical from 15 to 50 °C. This implies that elevated temperature does not induce the formation of imperfections in the wax barrier of *R. stricta* and, therefore, does not negatively affect the efficacy of the cuticular transpiration barrier. We argue that the reason for this unusual behaviour of *R. stricta* be the reinforcement of the cutin matrix by extensive deposits of triterpenoids acting as fillers and thus protecting the cutin polymer against a thermal expansion exceeding that of the waxes forming the diffusion barrier. Obviously, this is very advantageous for this plant growing in a very hot and arid habitat.

## Conclusions

In conclusion, based on the results and interpretations of this work with the representative hot-desert shrub *R. stricta*, the cuticle, its composition and water permeability in the future may be considered in a broader ecological context. Unfortunately, this is currently only rarely the case. Cuticular features may be recognized as relevant contributions to plant traits ensuring the competitiveness and even survival of a plant under adverse environmental conditions, especially under heat and drought stress. The extent and time to which sufficient amounts of water are available in the photosynthesizing parts largely depend on the efficacy of the control of water loss at maximum stomatal closure. Moreover, this efficacy essentially depends on cuticular water permeability. However, it is not only cuticular permeability *per se* which is important. In all species exposed to pronounced levels and rapid changes in temperature, the thermal stability of the cuticular transpiration barrier is decisive, maybe even to a higher degree than baseline cuticular permeability.

## Sources of Funding

This project was funded by the National Plan for Science, Technology and Innovation (MAARIFAH), King Abdulaziz City for Science and Technology, Kingdom of Saudi Arabia, Award Number (12-ENV2564-02). A.B. was supported by a scholarship from Capes Foundation, Ministry of Education of Brazil, Process number: 8908-13-3. This work was also supported by a Chinese Academy of Sciences Visiting Professorship for Senior International Scientists grant no. 2011T2S31 to M.R.

## Contributions by Authors

M.R., M.B. and R.H. conceived the research. A.-C.S., M.R., M.B., J.T., A.B. and J.L. designed and performed the experiments and analysed the results. A.-C.S. wrote the first draft and M.R., M.B. and J.L. contributed to revisions. All authors read and approved the manuscript.

## Conflicts of Interest Statement

None declared.

## Supplementary Material

Supplementary Data

## Supporting Information

The following additional information is available in the online version of this article —

**File 1.** Use and physical meaning of the terms cuticular permeability, minimum conductance and permeance.

**File 2.** Morphological leaf traits.

**File3.** Leaf water potential and pressure-volume analysis.

**File 4.** Leaf thermal and hydric tolerances.

**File 5.** Literature data used to test whether the leaf cuticular water permeability of *R.* *stricta* is lower than that of woody species from non-desert habitats. The median plus upper and lower confidence limits (*P* = 0.05) of cuticular permeances or minimum leaf conductances (*g*_min_) of 12 deciduous woody plant species (21 data points), the median plus upper and lower confidence limits (*P* = 0.05) of 13 evergreen woody plant species (44 data points), an average minimum conductance for seven unspecified evergreen desert shrubs and the mean minimum conductances of 2 desert grasses (3 data points) are documented.

## References

[plw027-B1] Abd El-Ghani MM. 1997 Phenology of ten common plant species in Western Saudi Arabia. Journal of Arid Environments 35:673–683.

[plw027-B2] Al-Ghobari HM. 2000 Estimation of reference evapotranspiration for Southern region of Saudi Arabia. Irrigation Science 19:81–86.

[plw027-B72] Al-Khamis HH Al-Hemaid FM Ibrahim ASS 2012 Diversity of perennial plants at Ibex Reserve in Saudi Arabia. The Journal of Animal and Plant Sciences 22:484–492.

[plw027-B3] Almazroui M Nazrul Islam M Jones PD Athar H Ashfaqur Rahman M. 2012 Recent climate change in the Arabian Peninsula: seasonal rainfall and temperature climatology of Saudi Arabia for 1979 – 2009. Atmospheric Research 111:29–45.

[plw027-B4] Baker EA. 1982 Chemistry and morphology of plant epicuticular waxes In: Cutler DF Alvin KL Price CE, eds. The plant cuticle. London: Academic Press, 139–165.

[plw027-B5] Burghardt M Riederer M. 2003 Ecophysiological relevance of cuticular transpiration of deciduous and evergreen plants in relation to stomatal closure and leaf water potential. Journal of Experimental Botany 54:1941–1949. 12815029 10.1093/jxb/erg195

[plw027-B73] Burghardt M Riederer M. 2006 Cuticular transpiration. In: Riederer M Müller C, eds. Biology of the plant cuticle, Vol. 23 Oxford: Blackwell Publishing, 292–311.

[plw027-B6] Buschhaus C Herz H Jetter R. 2007 Chemical composition of the epicuticular and intracuticular wax layers on the adaxial side of *Ligustrum vulgare* leaves. New Phytologist 176:311–316. 17696977 10.1111/j.1469-8137.2007.02190.x

[plw027-B7] Buschhaus C Jetter R. 2011 Composition differences between epicuticular and intracuticular wax substructures: how do plants seal their epidermal surfaces? Journal of Experimental Botany 62:841–853. 21193581 10.1093/jxb/erq366

[plw027-B8] Buschhaus C Jetter R. 2012 Composition and physiological function of the wax layers coating *Arabidopsis* leaves: β-amyrin negatively affects the intracuticular water barrier. Plant Physiology 160:1120–1129. 22885935 10.1104/pp.112.198473 PMC3461534

[plw027-B9] Bush RT McInerney FA. 2015 Influence of temperature and C_4_ abundance on *n*-alkane chain length distributions across the central USA. Organic Geochemistry 79:65–73.

[plw027-B10] Casado CG Heredia A. 1999 Structure and dynamics of reconstituted cuticular waxes of grape berry cuticle (*Vitis vinifera* L.). Journal of Experimental Botany 50:175–182.

[plw027-B11] Deil U Al-Gifri A-N. 1998 Montane and Wadi vegetation In: Ghazanfar SA Fisher M, eds. Vegetation of the Arabian Peninsula. Dordrecht: Kluwer Academic Publishers, 125–174.

[plw027-B12] De Micco V Aronne G. 2012 Morpho-anatomical traits for plant adaptation to drought In: Aroca R, ed. Plant responses to drought stress. Berlin: Springer, 37–61.

[plw027-B13] Dragota S Riederer M. 2008 Comparative study on epicuticular leaf waxes of *Araucaria araucana, Agathis robusta* and *Wollemia nobilis* (Araucariaceae). Australian Journal of Botany 56:644–650.

[plw027-B14] Eckl K Gruler H. 1980 Phase transitions in plant puticles. Planta 150:102–113. 24306583 10.1007/BF00582352

[plw027-B15] Emad El-Deen HM. 2005 Population ecology of *Rhazya stricta* Decne. in Western Saudi Arabia. International Journal of Agriculture and Biology 7:932–938.

[plw027-B16] España L Heredia-Guerrero JA Segado P Benítez JJ Heredia A Domínguez E. 2014 Biomechanical properties of the tomato (*Solanum lycopersicum*) fruit cuticle during development are modulated by changes in the relative amounts of its components. New Phytologist 202:790–802. 24571168 10.1111/nph.12727

[plw027-B17] Gibbs AG Louie AK Ayala JA. 1998 Effects of temperature on cuticular lipids and water balance in a desert *Drosophila*: is thermal acclimation beneficial? Journal of Experimental Biology 201:71–80. 9390938 10.1242/jeb.201.1.71

[plw027-B18] Grace J. 1989 Measurement of wind speed near vegetation In: Pearcy RW Ehleringer JR Mooney HA Rundel PW, eds. Plant physiological ecology, field methods and instrumentation. New York: Chapman and Hall, 57–73.

[plw027-B19] Goodwin SM Jenks MA. 2005 Plant cuticle function as a barrier to water loss In: Jenks MA Hasegawa PM, eds. Plant abiotic stress. Oxford: Blackwell Publishing, 14–36.

[plw027-B20] Hadley NF. 1972 Desert species and adaptation. American Scientist 60:338–347. 5064210

[plw027-B21] Hadley NF. 1981 Cuticular lipids of terrestrial plants and arthropods: a comparison of their structure, composition, and waterproofing function. Biological Reviews 56:23–47.

[plw027-B22] Hadley NF. 1989 Lipid water barriers in biological systems. Progress in Lipid Research 28:1–33. 2682668 10.1016/0163-7827(89)90005-2

[plw027-B23] Holloway PJ. 1982 The chemical constitution of plant cutins In: Cutler DF Alvin KL Price CE, eds. The plant cuticle. London: Academic Press, 45–85.

[plw027-B24] Holloway PJ. 1984 Cutins and suberins, the polymeric plant lipids In: Mangold HK Zweig G Sherma J, eds. CRC handbook of chromatography, lipids, *Vol* 1 Boca Raton: CRC Press, 321–345.

[plw027-B25] Holz M Heil SR Sacco A. 2000 Temperature-dependent self-diffusion coefficients of water and six selected molecular liquids for calibration in accurate ^1^H NMR PFG measurements. Physical Chemistry Chemical Physics 2:4740–4742.

[plw027-B26] Jeffree CE. (2006) The fine structure of the plant cuticle In: Riederer M Müller C, eds. Biology of the plant cuticle. Oxford, UK: Blackwell Publishing, 11–125.

[plw027-B27] Jetter R Schäffer S Riederer M. 2000 Leaf cuticular waxes are arranged in chemically and mechanically distinct layers: evidence from *Prunus laurocerasus* L. Plant, Cell and Environment 23:619–628.

[plw027-B28] Jetter R Kunst L Samuels AL. 2006 Composition of plant cuticular waxes In: Riederer M Müller C, eds. Biology of the plant cuticle, annual plant reviews, Vol. 23 Oxford: Blackwell Publishing, 145–181.

[plw027-B29] Jetter R Riederer M. 2016 Localization of the transpiration barrier in the epi- and intracuticular waxes of eight plant species: water transport resistances are associated with fatty acyl rather than alicyclic components. Plant Physiology 170:921–934. 26644508 10.1104/pp.15.01699 PMC4734581

[plw027-B30] Jones HG. 2013 Plants and microclimate: a quantitative approach to environmental plant physiology. Cambridge: Cambridge University Press pp 147, 261, 262.

[plw027-B31] Kerstiens G. 2006 Water transport in plant cuticles: an update. Journal of Experimental Botany 57:2493–2499. 16822810 10.1093/jxb/erl017

[plw027-B32] Körner C. 1994 Scaling from species to vegetation: the usefulness of functional groups In: Schulze ED Mooney HA, eds. Biodiversity and ecosystem function. Berlin: Springer, 117–140.

[plw027-B33] Körner C. 1995 Leaf diffusive conductances in the major vegetation types of the globe In: Schulze ED Caldwell MM, eds. Ecophysiology of photosynthesis. Berlin: Springer, 463–490.

[plw027-B34] Krishna LV. 2014 Long term temperature trends in four different climatic zones of Saudi Arabia. International Journal of Applied Science and Technology 4:233–242.

[plw027-B35] Lawson T Davey PA Yates SA Bechtold U Baeshen M Baeshen N Mutwakil MZ Sabir J Baker NR Mullineaux PM. 2013 C_3_ photosynthesis in the desert plant *Rhazya stricta* is fully functional at high temperatures and light intensities. New Phytologist 201:862–873. 24164092 10.1111/nph.12559

[plw027-B36] Leide J Hildebrandt U Reussing K Riederer M Vogg G. 2007 The developmental pattern of tomato fruit wax accumulation and its impact on cuticular transpiration barrier properties: effects of a deficiency in a beta-ketoacyl-coenzyme A synthase (LeCER6). Plant Physiolgy 144:1667–1679. 10.1104/pp.107.099481 PMC1914139 17468214

[plw027-B37] Leide J Hildebrandt U Vogg G Riederer M. 2011 The positional sterile (ps) mutation affects cuticular transpiration and wax biosynthesis of tomato fruits. Journal of Plant Physiology 168:871–877. 21242016 10.1016/j.jplph.2010.11.014

[plw027-B38] Lüttge U. 2007 Physiological ecology of tropical plants. Berlin: Springer Science & Business Media, 328.

[plw027-B39] Markstädter C Federle W Jetter R Riederer M Hölldobler B. 2000 Chemical composition of the slippery epicuticular wax blooms on *Macaranga* (Euphorbiaceae) ant-plants. Chemoecology 10:33–40.

[plw027-B40] McCrum NG Buckley CP Bucknall CB. 1997 Principles of polymer engineering. Oxford: Oxford University Press.

[plw027-B41] Merk S Blume A Riederer M. 1998 Phase behaviour and crystallinity of plant cuticular waxes studied by Fourier transform infrared spectroscopy. Planta 204:44–53.

[plw027-B42] Nobel PS. 2009 Physicochemical and environmental plant physiology, 4th edn Oxford: Academic Press.

[plw027-B44] Pearcy RW Ehleringer JR Mooney HA Rundel PW. 1989 Plant physiological ecology, field methods and instrumentation.London, UK: Chapman and Hall.

[plw027-B45] Pollard M Beisson F Li Y Ohlrogge JB. 2008 Building lipid barriers: biosynthesis of cutin and suberin. Trends in Plant Science 13:236–246. 18440267 10.1016/j.tplants.2008.03.003

[plw027-B46] Poorter H Garnier E. 2007 Ecological significance of inherent variation in relative growth rate and its components In: Pugnaire FI Valladares F, eds. Functional plant ecology. Boca Raton: CRC Press, 67–100.

[plw027-B47] Purves WK Sadava D Orians GH Heller HC. 2004 Life: the science of biology. 7th ed. Sunderland: Sinauer Associates, 772.

[plw027-B48] Riederer M Schneider G. 1990 The effect of the environment on the permeability and composition of *Citrus* leaf cuticles: 2. Composition of soluble cuticular lipids and correlation with transport properties. Planta 180:154–165. 24201939 10.1007/BF00193990

[plw027-B49] Riederer M Schreiber L. 1995 Waxes—the transport barriers of plant cuticles In: Hamilton RJ, ed. Waxes: chemistry, molecular biology and functions. Dundee: The Oily Press, 131–156.

[plw027-B50] Riederer M Schreiber L. 2001 Protecting against water loss: analysis of the barrier properties of plant cuticles. Journal of Experimental Botany 52:2023–2032. 11559738 10.1093/jexbot/52.363.2023

[plw027-B51] Riederer M. 2006 Thermodynamics of the water permeability of plant cuticles: characterization of the polar pathway. Journal of Experimental Botany 57:2937–2942. 16873453 10.1093/jxb/erl053

[plw027-B52] Schönherr J. 1976 Water permeability of isolated cuticular membranes: the effect of cuticular waxes on diffusion of water. Planta 131:159–164. 24424766 10.1007/BF00389989

[plw027-B53] Schönherr J Lendzian K. 1981 A simple and inexpensive method of measuring water permeability of isolated plant cuticular membranes. Zeitschrift für Pflanzenphysiologie 102:321–327.

[plw027-B54] Schönherr J. 1982 Resistance of plant surfaces to water loss: transport properties of cutin, suberin and associated lipids In: Lange OL Nobel PS Osmond CB Ziegler H, eds. Encyclopedia of plant physiology. Physiological plant ecology II, Water relations and carbon assimilation, Vol. 12B. Berlin: Springer, 153–179.

[plw027-B55] Schönherr J Riederer M. 1986 Plant cuticles sorb lipophilic compounds during enzymatic isolation. Plant, Cell and Environment 9:459–466.

[plw027-B56] Schreiber L Schönherr J. 1990 Phase transitions and thermal expansion coefficients of plant cuticles. Planta 182:186–193. 24197094 10.1007/BF00197109

[plw027-B57] Schreiber L Riederer M. 1996 Ecophysiology of cuticular transpiration: comparative investigation of cuticular water permeability of plant species from different habitats. Oecologia 107:426–432. 10.1007/BF00333931 28307383

[plw027-B58] Schreiber L. 2001 Effect of temperature on cuticular transpiration of isolated cuticular membranes and leaf discs. Journal of Experimental Botany 52:1893–1900. 11520878 10.1093/jexbot/52.362.1893

[plw027-B59] Slavík B. 1974 Methods of studying plant water relations. Berlin: Springer.

[plw027-B60] Smirnova A Leide J Riederer M. 2013 Analysis of flower cuticular waxes and cutin monomers. Bio-Protocol 3:e899.

[plw027-B61] Smith SD Monson RK Anderson JE. 2012 Physiological ecology of North American desert plants. Berlin: Springer Science & Business Media pp 229, 231.

[plw027-B62] Smith SE Fendenheim DM Halbrook K. 2006 Epidermal conductance as a component of dehydration avoidance in *Digitaria californica* and *Eragrostis lehmanniana*, two perennial desert grasses. Journal of Arid Environments 64:238–250.

[plw027-B63] Smith WK. 1978 Temperatures of desert plants: another perspective on the adaptability of leaf size. Science 201:614–616. 17794122 10.1126/science.201.4356.614

[plw027-B64] Stein WD. 1986 Transport and diffusion across cell membranes. New York, Academic Press.

[plw027-B65] Szakiel A Pączkowski C Pensec F Bertsch C. 2012 Fruit cuticular waxes as a source of biologically active triterpenoids. Phytochemistry Reviews 11:1–22. 10.1007/s11101-012-9241-9 PMC3601259 23519009

[plw027-B66] Tsubaki S Sugimura K Teramoto Y Yonemori K Azuma J. 2013 Cuticular membrane of *Fuyu* persimmon fruit is strengthened by triterpenoid nano-fillers. PLoS One 8:e75275. 24086493 10.1371/journal.pone.0075275 PMC3782500

[plw027-B67] Vogg G Fischer S Leide J Emmanuel E Jetter R Levy AA Riederer M. 2004 Tomato fruit cuticular waxes and their effects on transpiration barrier properties: functional characterization of a mutant deficient in a very-long-chain fatty acid β-ketoacyl-CoA synthase. Journal of Experimental Botany 55:1401–1410. 15133057 10.1093/jxb/erh149

[plw027-B68] Windholz M Budavari S Blumetti RF Otterbein ES. 1983 The Merck Index. New York: Merck & Co.

[plw027-B69] Wissemann V Riedel M Riederer M. 2007 Matroclinal inheritance of cuticular waxes in reciprocal hybrids of *Rosa* species, sect. *Caninae* (Rosaceae). Plant Systematics and Evolution 263:181–190.

[plw027-B70] Yates SA Chernukhin I Álvarez-Fernández R Bechtold U Baeshen M Baeshen N Mutwakil MZ Sabir J Lawson T Mullineaux PM. 2014 The temporal foliar transcriptome of the perennial C_3_ desert plant *Rhazya stricta* in its natural environment. BMC Plant Biology 14:2. 24387666 10.1186/1471-2229-14-2 PMC3906910

[plw027-B71] Xanthos M. 2010 Modification of polymer properties with functional fillers In: Xanthos M, ed. Functional fillers for plastics. New Jersey: Wiley & Sons, 19–41.

