# Supplementary material for: Effectiveness of cuticular transpiration barriers in a desert plant at controlling water loss at high temperatures
Source: AoB Plants. 2016 May 6;8:plw027. doi: 10.1093/aobpla/plw027 (PMC4925923; doi:10.1093/aobpla/plw027)
Supplement: Supplementary Data [file supp_8_plw027_index.html]

Effectiveness of cuticular transpiration barriers in a desert plant at controlling water loss at high temperatures — Effectiveness of cuticular transpiration barriers in a desert plant at controlling water loss at high temperatures — Supplementary Data 

# Effectiveness of cuticular transpiration barriers in a desert plant at controlling water loss at high temperatures

## Supplementary Data

files

- Supplementary Data - doc file
- Supplementary Data - pptx file
- Supplementary Data - docx file
- Supplementary Data - docx file
- Supplementary Data - docx file
