# Supplementary material for: Effectiveness of cuticular transpiration barriers in a desert plant at controlling water loss at high temperatures
Source: AoB Plants. 2016 May 6;8:plw027. doi: 10.1093/aobpla/plw027 (PMC4925923; doi:10.1093/aobpla/plw027)
Supplement: Supplementary Data [file supp_plw027_aobplants-15257-s_2.doc]

# File 4. Leaf thermal and hydric tolerances

### Material and methods

Chlorophyll fluorescence was used to determine the thermal and hydric tolerance limits of the leaves. Chlorophyll fluorescence is a widely-used technique to measure the ability of leaves to tolerate environmental stresses. The most frequently applied parameter is the maximum quantum yield of photosystem ll in the dark adapted state (F_v_/F_m_), since it is easy and fast to determine. The decline of F_v_/F_m_ indicates the occurrence of damage to the photosynthetic apparatus (Maxwell and Johnson 2000; Lichtenthaler *et al.* 2005) and provides an appropriate method to assess the limits of whole leaf survival in response to high temperature stress and drought stress. The decline of F_v_/F_m_ below 50 % of the maximum value of unstressed leaves can be taken as threshold for viability with the onset of irreversible damage (Woo *et al.* 2008, Curtis *et al.* 2014). The maximum quantum yield of photosystem ll in the dark-adapted state (F_v_/F_m_) was measured with a pulse-amplitude modulated fluorometer (measuring light: 5 Hz, actinic light: switched off, saturation pulse: intensity level 12 (10,000 µmol m^-2^ s^-1^), 0.6 seconds pulse width, Junior PAM, Walz, Effeltrich, Germany). The leaves were exposed to temperature treatments in the range from 25.0° to 65.0° C at 2.5 K intervals in the dark. The leaves were enclosed in plastic bags with moist filter paper and placed for 30 min in an incubator with precise temperature control (IPP110, setting accuracy 0.1 K, Memmert, Schwabach, Germany). F_v_/F_m_ was measured after recovery for 30 min at room temperature in the dark. Fresh samples were used for each temperature level. Two threshold temperatures for leaf thermal tolerance were determined. T_c_ is the critical temperature where F_v_/F_m_ starts to decrease strongly (Brestič and Živčák 2013). The non-stressed F_v_/F_m_ was calculated as the mean for 25.0° to 37.5° C. A quadratic function was fitted to the data for 47.5° to 60.0° C. The critical temperature (T_c_), indicating the onset of a substantial decrease of F_v_/F_m_, was identified by solving the equation for the temperature at the mean non-stressed F_v_/F_m_. As an additional stress indicator, T_50_ was determined which is the temperature where F_v_/F_m_ is reduced to 50% of the maximum at the non-stressed level (Knight and Ackerly 2003; Curtis *et al.* 2014). T_50_ was obtained equivalently to T_c_ by solving the equation for the temperature at half the mean non-stressed F_v_/F_m_.

For the measurement of the hydric tolerance, saturated leaves were progressively dried on the bench in the dark, and F_v_/F_m_ was measured for the different dehydration levels. The critical relative water deficit (RWD_c_) and the relative water deficit at 50% of the maximum value for the water saturated status (RWD_50_) were determined as described above for the estimation of T_c_ and T_50_. The non-stressed F_v_/F_m_ was calculated as the mean for 0.00 to 0.45 RWD. A linear function was fitted to the data for 0.55 to 1.00 RWD.

### Results

The leaf thermal and hydric tolerances were established to delimit the ecophysiologically allowable ranges of temperature and dehydration to be used in experimentation. The maximum quantum yield of photosystem ll in the dark-adapted state (F_v_/F_m_) was measured as an easily accessible proxy for leaf viability. F_v_/F_m_ of *R. stricta* leaves was only slightly affected by temperature from 25° to 42.5° C, while F_v_/F_m_ decreased strongly at temperatures above 45° C (Fig. S3). The estimate for the critical temperature (T_c_) was 46.1° C, indicating the onset of the decreasing branch of F_v_/F_m_. The temperature at which F_v_/F_m_ declined to 50% of the initial maximum at the non-heat-stressed level (T_50_) was 50.8° C. During leaf dehydration, F_v_/F_m_ remained high down to a RWD of slightly less than 0.5. At higher RWD values however, F_v_/F_m_ started to decline (Fig. S4). The estimated critical RWD (RWD_c_) was 0.59 indicating the onset of the decreasing branch of F_v_/F_m_. The RWD at which F_v_/F_m_ declined to 50% of the initial maximum at the non-drought-stressed level (RWD_50_) was 0.78.
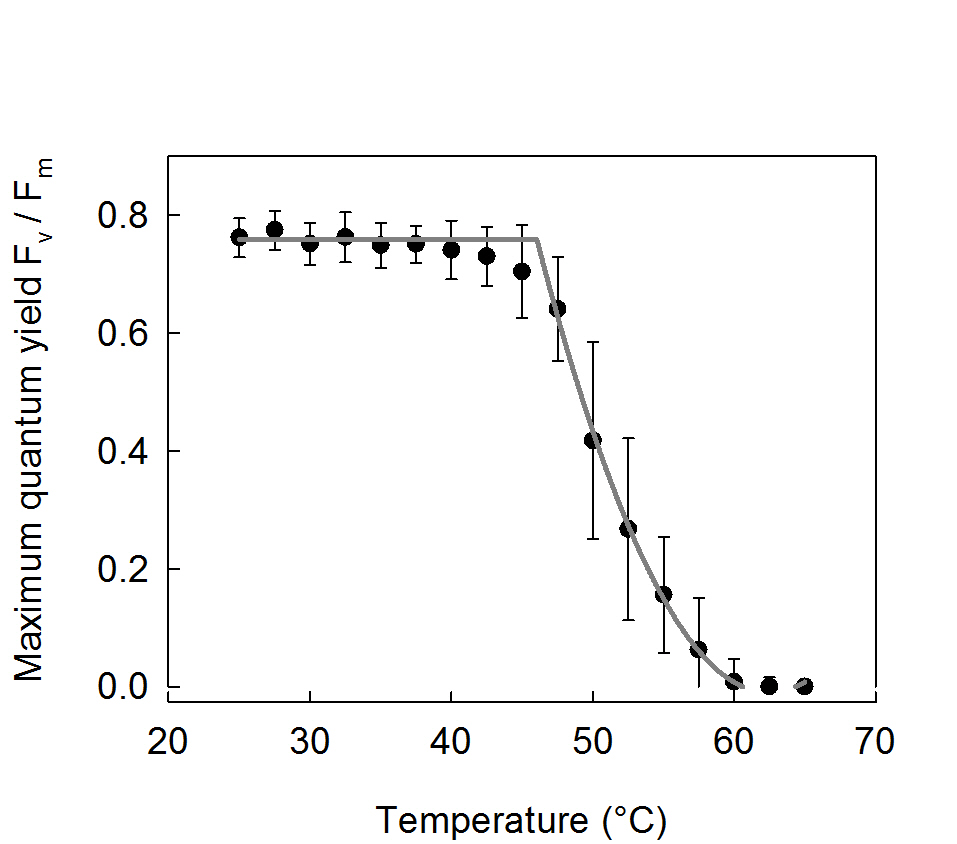


**Figure S3.** Effect of pre-treatment temperature on the maximum quantum yield of photosystem ll of leaves in the dark-adapted state (F_v_/F_m_) subsequently measured at room temperature. Each point represents the mean value ± SD (n ≥ 9). The mean value for the temperature range between 25.0° C and 42.5° C and the graph for the regression between 45° and 55° C are shown.


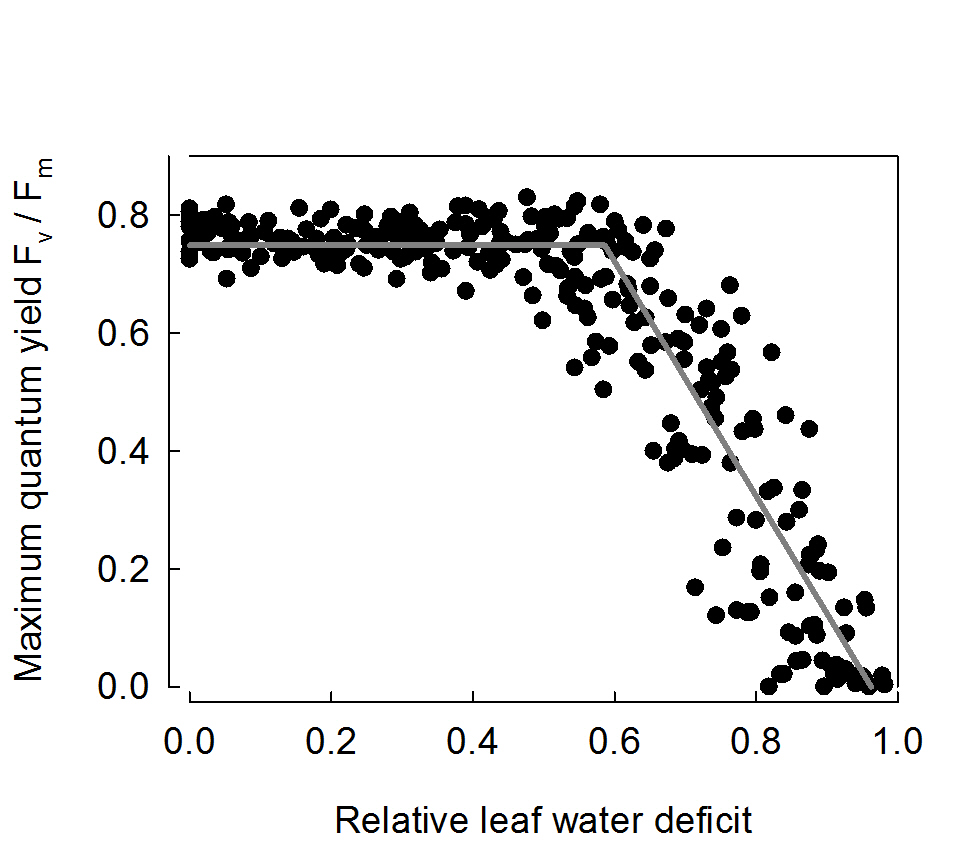


**Figure S4.** The maximum quantum yield of photosystem ll in the dark-adapted state (F_v_/F_m_) during leaf dehydration as a function of relative water deficit (RWD). Each point represents an individual measurement obtained from dehydration experiments with a total of 18 leaves. The mean value for RWD ≤ 0.6 and the graph for the regression for higher RWD values are shown.

### Literature

Brestič M, Živčák M. 2013. PSll fluorescence techniques for measurement of drought and high temperature stress signal in crop plants: protocols and application. In: Rout GR, Das GR, eds. *Molecular stress physiology of plants*. Berlin: Springer-Verlag, 97-131.

Curtis EM, Knight CA, Petrou K, Leigh A. 2014. A comparative analysis of photosynthetic recovery from thermal stress: a desert plant case study. *Oecologia* 175:1051-1061.

Knight CA, Ackerly DD. 2003. Evolution and plasticity of photosynthetic thermal tolerance, specific leaf area and leaf size: congeneric species from desert and coastal environments. *New Phytologist* 160:337-347.

Lichtenthaler HK, Buschmann C, Knapp M. 2005. How to correctly determine the different chlorophyll fluorescence parameters and the chlorophyll fluorescence decrease ratio R_Fd_ of leaves with the PAM fluorometer. *Photosynthetica* 43:379-393.

Maxwell K, Johnson GN. 2000. Chlorophyll fluorescence – a practical guide. *Journal of Experimental Botany* 51:659-668.

Woo NS, Badger MR, Pogson BJ. 2008. A rapid, non-invasive procedure for quantitative assessment of drought survival using chlorophyll fluorescence. *Plant Methods* 4:27.
