# Supplementary material for: Effectiveness of cuticular transpiration barriers in a desert plant at controlling water loss at high temperatures
Source: AoB Plants. 2016 May 6;8:plw027. doi: 10.1093/aobpla/plw027 (PMC4925923; doi:10.1093/aobpla/plw027)
Supplement: Supplementary Data [file supp_plw027_aobplants-15257-s_3.pptx]

## Slide 1
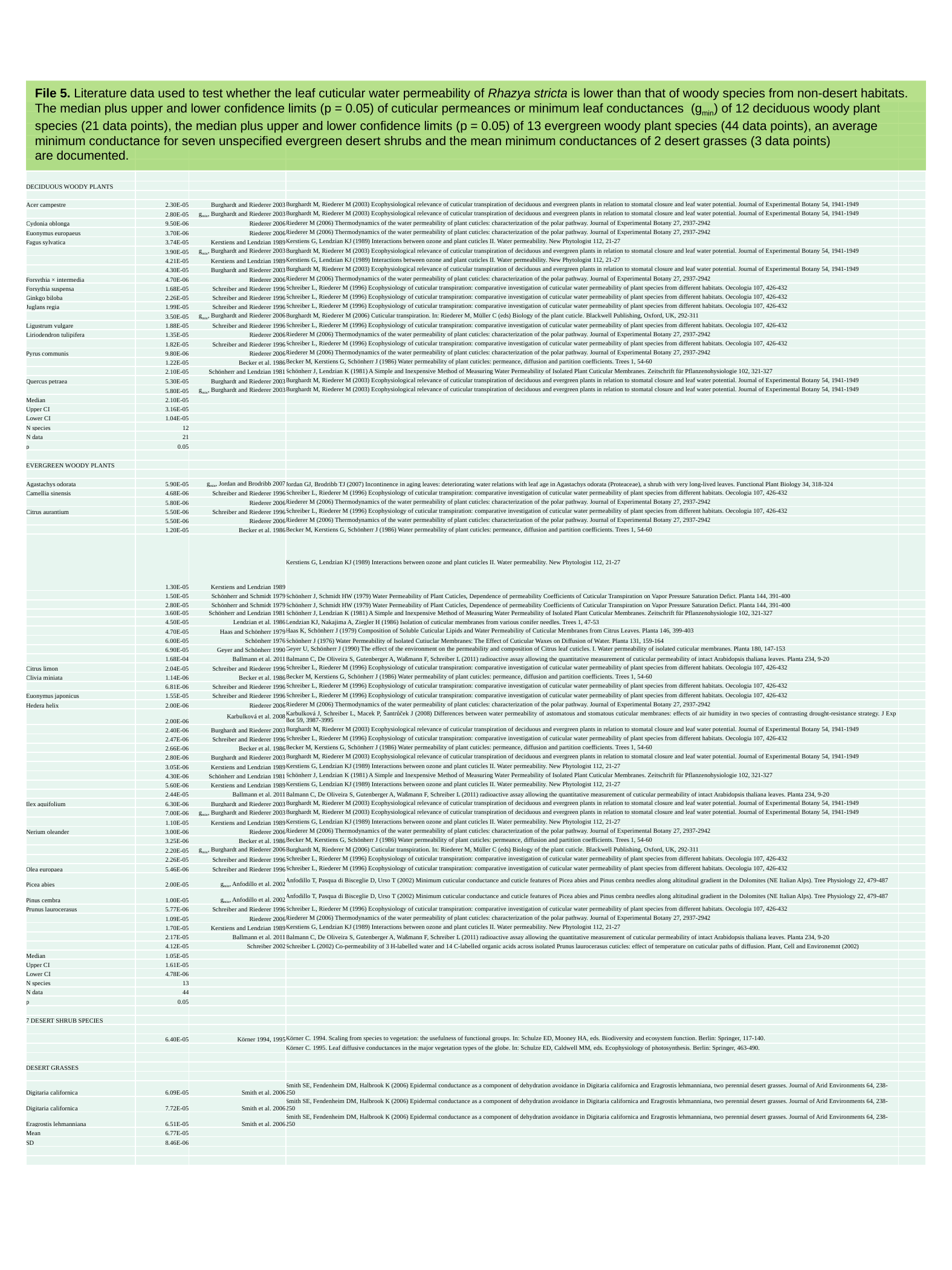

File 5. Literature data used to test whether the leaf cuticular water permeability of Rhazya stricta is lower than that of woody species from non-desert habitats.
The median plus upper and lower confidence limits (p = 0.05) of cuticular permeances or minimum leaf conductances (gmin) of 12 deciduous woody plant
species (21 data points), the median plus upper and lower confidence limits (p = 0.05) of 13 evergreen woody plant species (44 data points), an average
minimum conductance for seven unspecified evergreen desert shrubs and the mean minimum conductances of 2 desert grasses (3 data points)
are documented.
| | | | | |
| --- | --- | --- | --- | --- |
| | | | | |
| | | | | |
| | | | | |
| | | | | |
| | | | | |
| | | | | |
| DECIDUOUS WOODY PLANTS | | | | |
| | | | | |
| Acer campestre | 2.30E-05 | Burghardt and Riederer 2003 | Burghardt M, Riederer M (2003) Ecophysiological relevance of cuticular transpiration of deciduous and evergreen plants in relation to stomatal closure and leaf water potential. Journal of Experimental Botany 54, 1941-1949 | |
| | 2.80E-05 | gmin, Burghardt and Riederer 2003 | Burghardt M, Riederer M (2003) Ecophysiological relevance of cuticular transpiration of deciduous and evergreen plants in relation to stomatal closure and leaf water potential. Journal of Experimental Botany 54, 1941-1949 | |
| Cydonia oblonga | 9.50E-06 | Riederer 2006 | Riederer M (2006) Thermodynamics of the water permeability of plant cuticles: characterization of the polar pathway. Journal of Experimental Botany 27, 2937-2942 | |
| Euonymus europaeus | 3.70E-06 | Riederer 2006 | Riederer M (2006) Thermodynamics of the water permeability of plant cuticles: characterization of the polar pathway. Journal of Experimental Botany 27, 2937-2942 | |
| Fagus sylvatica | 3.74E-05 | Kerstiens and Lendzian 1989 | Kerstiens G, Lendzian KJ (1989) Interactions between ozone and plant cuticles II. Water permeability. New Phytologist 112, 21-27 | |
| | 3.90E-05 | gmin, Burghardt and Riederer 2003 | Burghardt M, Riederer M (2003) Ecophysiological relevance of cuticular transpiration of deciduous and evergreen plants in relation to stomatal closure and leaf water potential. Journal of Experimental Botany 54, 1941-1949 | |
| | 4.21E-05 | Kerstiens and Lendzian 1989 | Kerstiens G, Lendzian KJ (1989) Interactions between ozone and plant cuticles II. Water permeability. New Phytologist 112, 21-27 | |
| | 4.30E-05 | Burghardt and Riederer 2003 | Burghardt M, Riederer M (2003) Ecophysiological relevance of cuticular transpiration of deciduous and evergreen plants in relation to stomatal closure and leaf water potential. Journal of Experimental Botany 54, 1941-1949 | |
| Forsythia × intermedia | 4.70E-06 | Riederer 2006 | Riederer M (2006) Thermodynamics of the water permeability of plant cuticles: characterization of the polar pathway. Journal of Experimental Botany 27, 2937-2942 | |
| Forsythia suspensa | 1.68E-05 | Schreiber and Riederer 1996 | Schreiber L, Riederer M (1996) Ecophysiology of cuticular transpiration: comparative investigation of cuticular water permeability of plant species from different habitats. Oecologia 107, 426-432 | |
| Ginkgo biloba | 2.26E-05 | Schreiber and Riederer 1996 | Schreiber L, Riederer M (1996) Ecophysiology of cuticular transpiration: comparative investigation of cuticular water permeability of plant species from different habitats. Oecologia 107, 426-432 | |
| Juglans regia | 1.99E-05 | Schreiber and Riederer 1996 | Schreiber L, Riederer M (1996) Ecophysiology of cuticular transpiration: comparative investigation of cuticular water permeability of plant species from different habitats. Oecologia 107, 426-432 | |
| | 3.50E-05 | gmin, Burghardt and Riederer 2006 | Burghardt M, Riederer M (2006) Cuticular transpiration. In: Riederer M, Müller C (eds) Biology of the plant cuticle. Blackwell Publishing, Oxford, UK, 292-311 | |
| Ligustrum vulgare | 1.88E-05 | Schreiber and Riederer 1996 | Schreiber L, Riederer M (1996) Ecophysiology of cuticular transpiration: comparative investigation of cuticular water permeability of plant species from different habitats. Oecologia 107, 426-432 | |
| Liriodendron tulipifera | 1.35E-05 | Riederer 2006 | Riederer M (2006) Thermodynamics of the water permeability of plant cuticles: characterization of the polar pathway. Journal of Experimental Botany 27, 2937-2942 | |
| | 1.82E-05 | Schreiber and Riederer 1996 | Schreiber L, Riederer M (1996) Ecophysiology of cuticular transpiration: comparative investigation of cuticular water permeability of plant species from different habitats. Oecologia 107, 426-432 | |
| Pyrus communis | 9.80E-06 | Riederer 2006 | Riederer M (2006) Thermodynamics of the water permeability of plant cuticles: characterization of the polar pathway. Journal of Experimental Botany 27, 2937-2942 | |
| | 1.22E-05 | Becker et al. 1986 | Becker M, Kerstiens G, Schönherr J (1986) Water permeability of plant cuticles: permeance, diffusion and partition coefficients. Trees 1, 54-60 | |
| | 2.10E-05 | Schönherr and Lendzian 1981 | Schönherr J, Lendzian K (1981) A Simple and Inexpensive Method of Measuring Water Permeability of Isolated Plant Cuticular Membranes. Zeitschrift für Pflanzenohysiologie 102, 321-327 | |
| Quercus petraea | 5.30E-05 | Burghardt and Riederer 2003 | Burghardt M, Riederer M (2003) Ecophysiological relevance of cuticular transpiration of deciduous and evergreen plants in relation to stomatal closure and leaf water potential. Journal of Experimental Botany 54, 1941-1949 | |
| | 5.80E-05 | gmin, Burghardt and Riederer 2003 | Burghardt M, Riederer M (2003) Ecophysiological relevance of cuticular transpiration of deciduous and evergreen plants in relation to stomatal closure and leaf water potential. Journal of Experimental Botany 54, 1941-1949 | |
| Median | 2.10E-05 | | | |
| Upper CI | 3.16E-05 | | | |
| Lower CI | 1.04E-05 | | | |
| N species | 12 | | | |
| N data | 21 | | | |
| p | 0.05 | | | |
| | | | | |
| EVERGREEN WOODY PLANTS | | | | |
| | | | | |
| Agastachys odorata | 5.90E-05 | gmin, Jordan and Brodribb 2007 | Jordan GJ, Brodribb TJ (2007) Incontinence in aging leaves: deteriorating water relations with leaf age in Agastachys odorata (Proteaceae), a shrub with very long-lived leaves. Functional Plant Biology 34, 318-324 | |
| Camellia sinensis | 4.68E-06 | Schreiber and Riederer 1996 | Schreiber L, Riederer M (1996) Ecophysiology of cuticular transpiration: comparative investigation of cuticular water permeability of plant species from different habitats. Oecologia 107, 426-432 | |
| | 5.80E-06 | Riederer 2006 | Riederer M (2006) Thermodynamics of the water permeability of plant cuticles: characterization of the polar pathway. Journal of Experimental Botany 27, 2937-2942 | |
| Citrus aurantium | 5.50E-06 | Schreiber and Riederer 1996 | Schreiber L, Riederer M (1996) Ecophysiology of cuticular transpiration: comparative investigation of cuticular water permeability of plant species from different habitats. Oecologia 107, 426-432 | |
| | 5.50E-06 | Riederer 2006 | Riederer M (2006) Thermodynamics of the water permeability of plant cuticles: characterization of the polar pathway. Journal of Experimental Botany 27, 2937-2942 | |
| | 1.20E-05 | Becker et al. 1986 | Becker M, Kerstiens G, Schönherr J (1986) Water permeability of plant cuticles: permeance, diffusion and partition coefficients. Trees 1, 54-60 | |
| | 1.30E-05 | Kerstiens and Lendzian 1989 | Kerstiens G, Lendzian KJ (1989) Interactions between ozone and plant cuticles II. Water permeability. New Phytologist 112, 21-27 | |
| | 1.50E-05 | Schönherr and Schmidt 1979 | Schönherr J, Schmidt HW (1979) Water Permeability of Plant Cuticles, Dependence of permeability Coefficients of Cuticular Transpiration on Vapor Pressure Saturation Defict. Planta 144, 391-400 | |
| | 2.80E-05 | Schönherr and Schmidt 1979 | Schönherr J, Schmidt HW (1979) Water Permeability of Plant Cuticles, Dependence of permeability Coefficients of Cuticular Transpiration on Vapor Pressure Saturation Defict. Planta 144, 391-400 | |
| | 3.60E-05 | Schönherr and Lendzian 1981 | Schönherr J, Lendzian K (1981) A Simple and Inexpensive Method of Measuring Water Permeability of Isolated Plant Cuticular Membranes. Zeitschrift für Pflanzenohysiologie 102, 321-327 | |
| | 4.50E-05 | Lendzian et al. 1986 | Lendzian KJ, Nakajima A, Ziegler H (1986) Isolation of cuticular membranes from various conifer needles. Trees 1, 47-53 | |
| | 4.70E-05 | Haas and Schönherr 1979 | Haas K, Schönherr J (1979) Composition of Soluble Cuticular Lipids and Water Permeability of Cuticular Membranes from Citrus Leaves. Planta 146, 399-403 | |
| | 6.00E-05 | Schönherr 1976 | Schönherr J (1976) Water Permeability of Isolated Cutiuclar Membranes: The Effect of Cuticular Waxes on Diffusion of Water. Planta 131, 159-164 | |
| | 6.90E-05 | Geyer and Schönherr 1990 | Geyer U, Schönherr J (1990) The effect of the environment on the permeability and composition of Citrus leaf cuticles. I. Water permeability of isolated cuticular membranes. Planta 180, 147-153 | |
| | 1.68E-04 | Ballmann et al. 2011 | Balmann C, De Oliveira S, Gutenberger A, Waßmann F, Schreiber L (2011) radioactive assay allowing the quantitative measurement of cuticular permeability of intact Arabidopsis thaliana leaves. Planta 234, 9-20 | |
| Citrus limon | 2.04E-05 | Schreiber and Riederer 1996 | Schreiber L, Riederer M (1996) Ecophysiology of cuticular transpiration: comparative investigation of cuticular water permeability of plant species from different habitats. Oecologia 107, 426-432 | |
| Clivia miniata | 1.14E-06 | Becker et al. 1986 | Becker M, Kerstiens G, Schönherr J (1986) Water permeability of plant cuticles: permeance, diffusion and partition coefficients. Trees 1, 54-60 | |
| | 6.81E-06 | Schreiber and Riederer 1996 | Schreiber L, Riederer M (1996) Ecophysiology of cuticular transpiration: comparative investigation of cuticular water permeability of plant species from different habitats. Oecologia 107, 426-432 | |
| Euonymus japonicus | 1.55E-05 | Schreiber and Riederer 1996 | Schreiber L, Riederer M (1996) Ecophysiology of cuticular transpiration: comparative investigation of cuticular water permeability of plant species from different habitats. Oecologia 107, 426-432 | |
| Hedera helix | 2.00E-06 | Riederer 2006 | Riederer M (2006) Thermodynamics of the water permeability of plant cuticles: characterization of the polar pathway. Journal of Experimental Botany 27, 2937-2942 | |
| | 2.00E-06 | Karbulková et al. 2008 | Karbulková J, Schreiber L, Macek P, Šantrůček J (2008) Differences between water permeability of astomatous and stomatous cuticular membranes: effects of air humidity in two species of contrasting drought-resistance strategy. J Exp Bot 59, 3987-3995 | |
| | 2.40E-06 | Burghardt and Riederer 2003 | Burghardt M, Riederer M (2003) Ecophysiological relevance of cuticular transpiration of deciduous and evergreen plants in relation to stomatal closure and leaf water potential. Journal of Experimental Botany 54, 1941-1949 | |
| | 2.47E-06 | Schreiber and Riederer 1996 | Schreiber L, Riederer M (1996) Ecophysiology of cuticular transpiration: comparative investigation of cuticular water permeability of plant species from different habitats. Oecologia 107, 426-432 | |
| | 2.66E-06 | Becker et al. 1986 | Becker M, Kerstiens G, Schönherr J (1986) Water permeability of plant cuticles: permeance, diffusion and partition coefficients. Trees 1, 54-60 | |
| | 2.80E-06 | Burghardt and Riederer 2003 | Burghardt M, Riederer M (2003) Ecophysiological relevance of cuticular transpiration of deciduous and evergreen plants in relation to stomatal closure and leaf water potential. Journal of Experimental Botany 54, 1941-1949 | |
| | 3.05E-06 | Kerstiens and Lendzian 1989 | Kerstiens G, Lendzian KJ (1989) Interactions between ozone and plant cuticles II. Water permeability. New Phytologist 112, 21-27 | |
| | 4.30E-06 | Schönherr and Lendzian 1981 | Schönherr J, Lendzian K (1981) A Simple and Inexpensive Method of Measuring Water Permeability of Isolated Plant Cuticular Membranes. Zeitschrift für Pflanzenohysiologie 102, 321-327 | |
| | 5.60E-06 | Kerstiens and Lendzian 1989 | Kerstiens G, Lendzian KJ (1989) Interactions between ozone and plant cuticles II. Water permeability. New Phytologist 112, 21-27 | |
| | 2.44E-05 | Ballmann et al. 2011 | Balmann C, De Oliveira S, Gutenberger A, Waßmann F, Schreiber L (2011) radioactive assay allowing the quantitative measurement of cuticular permeability of intact Arabidopsis thaliana leaves. Planta 234, 9-20 | |
| Ilex aquifolium | 6.30E-06 | Burghardt and Riederer 2003 | Burghardt M, Riederer M (2003) Ecophysiological relevance of cuticular transpiration of deciduous and evergreen plants in relation to stomatal closure and leaf water potential. Journal of Experimental Botany 54, 1941-1949 | |
| | 7.00E-06 | gmin, Burghardt and Riederer 2003 | Burghardt M, Riederer M (2003) Ecophysiological relevance of cuticular transpiration of deciduous and evergreen plants in relation to stomatal closure and leaf water potential. Journal of Experimental Botany 54, 1941-1949 | |
| | 1.10E-05 | Kerstiens and Lendzian 1989 | Kerstiens G, Lendzian KJ (1989) Interactions between ozone and plant cuticles II. Water permeability. New Phytologist 112, 21-27 | |
| Nerium oleander | 3.00E-06 | Riederer 2006 | Riederer M (2006) Thermodynamics of the water permeability of plant cuticles: characterization of the polar pathway. Journal of Experimental Botany 27, 2937-2942 | |
| | 3.25E-06 | Becker et al. 1986 | Becker M, Kerstiens G, Schönherr J (1986) Water permeability of plant cuticles: permeance, diffusion and partition coefficients. Trees 1, 54-60 | |
| | 2.20E-05 | gmin, Burghardt and Riederer 2006 | Burghardt M, Riederer M (2006) Cuticular transpiration. In: Riederer M, Müller C (eds) Biology of the plant cuticle. Blackwell Publishing, Oxford, UK, 292-311 | |
| | 2.26E-05 | Schreiber and Riederer 1996 | Schreiber L, Riederer M (1996) Ecophysiology of cuticular transpiration: comparative investigation of cuticular water permeability of plant species from different habitats. Oecologia 107, 426-432 | |
| Olea europaea | 5.46E-06 | Schreiber and Riederer 1996 | Schreiber L, Riederer M (1996) Ecophysiology of cuticular transpiration: comparative investigation of cuticular water permeability of plant species from different habitats. Oecologia 107, 426-432 | |
| Picea abies | 2.00E-05 | gmin, Anfodillo et al. 2002 | Anfodillo T, Pasqua di Bisceglie D, Urso T (2002) Minimum cuticular conductance and cuticle features of Picea abies and Pinus cembra needles along altitudinal gradient in the Dolomites (NE Italian Alps). Tree Physiology 22, 479-487 | |
| Pinus cembra | 1.00E-05 | gmin, Anfodillo et al. 2002 | Anfodillo T, Pasqua di Bisceglie D, Urso T (2002) Minimum cuticular conductance and cuticle features of Picea abies and Pinus cembra needles along altitudinal gradient in the Dolomites (NE Italian Alps). Tree Physiology 22, 479-487 | |
| Prunus laurocerasus | 5.77E-06 | Schreiber and Riederer 1996 | Schreiber L, Riederer M (1996) Ecophysiology of cuticular transpiration: comparative investigation of cuticular water permeability of plant species from different habitats. Oecologia 107, 426-432 | |
| | 1.09E-05 | Riederer 2006 | Riederer M (2006) Thermodynamics of the water permeability of plant cuticles: characterization of the polar pathway. Journal of Experimental Botany 27, 2937-2942 | |
| | 1.70E-05 | Kerstiens and Lendzian 1989 | Kerstiens G, Lendzian KJ (1989) Interactions between ozone and plant cuticles II. Water permeability. New Phytologist 112, 21-27 | |
| | 2.17E-05 | Ballmann et al. 2011 | Balmann C, De Oliveira S, Gutenberger A, Waßmann F, Schreiber L (2011) radioactive assay allowing the quantitative measurement of cuticular permeability of intact Arabidopsis thaliana leaves. Planta 234, 9-20 | |
| | 4.12E-05 | Schreiber 2002 | Schreiber L (2002) Co-permeability of 3 H-labelled water and 14 C-labelled organic acids across isolated Prunus laurocerasus cuticles: effect of temperature on cuticular paths of diffusion. Plant, Cell and Environemnt (2002) | |
| Median | 1.05E-05 | | | |
| Upper CI | 1.61E-05 | | | |
| Lower CI | 4.78E-06 | | | |
| N species | 13 | | | |
| N data | 44 | | | |
| p | 0.05 | | | |
| | | | | |
| 7 DESERT SHRUB SPECIES | | | | |
| | | | | |
| | 6.40E-05 | Körner 1994, 1995 | Körner C. 1994. Scaling from species to vegetation: the usefulness of functional groups. In: Schulze ED, Mooney HA, eds. Biodiversity and ecosystem function. Berlin: Springer, 117-140. | |
| | | | Körner C. 1995. Leaf diffusive conductances in the major vegetation types of the globe. In: Schulze ED, Caldwell MM, eds. Ecophysiology of photosynthesis. Berlin: Springer, 463-490. | |
| | | | | |
| DESERT GRASSES | | | | |
| | | | | |
| Digitaria californica | 6.09E-05 | Smith et al. 2006 | Smith SE, Fendenheim DM, Halbrook K (2006) Epidermal conductance as a component of dehydration avoidance in Digitaria californica and Eragrostis lehmanniana, two perennial desert grasses. Journal of Arid Environments 64, 238-250 | |
| Digitaria californica | 7.72E-05 | Smith et al. 2006 | Smith SE, Fendenheim DM, Halbrook K (2006) Epidermal conductance as a component of dehydration avoidance in Digitaria californica and Eragrostis lehmanniana, two perennial desert grasses. Journal of Arid Environments 64, 238-250 | |
| Eragrostis lehmanniana | 6.51E-05 | Smith et al. 2006 | Smith SE, Fendenheim DM, Halbrook K (2006) Epidermal conductance as a component of dehydration avoidance in Digitaria californica and Eragrostis lehmanniana, two perennial desert grasses. Journal of Arid Environments 64, 238-250 | |
| Mean | 6.77E-05 | | | |
| SD | 8.46E-06 | | | |
| | | | | |
| | | | | |
