# Supplementary material for: Effectiveness of cuticular transpiration barriers in a desert plant at controlling water loss at high temperatures
Source: AoB Plants. 2016 May 6;8:plw027. doi: 10.1093/aobpla/plw027 (PMC4925923; doi:10.1093/aobpla/plw027)
Supplement: Supplementary Data [file supp_plw027_aobplants-15257-s_4.docx]

# **File 1. Use and physical meaning of the terms cuticular permeability, permeance and minimum conductance**

To improve readability, “cuticular permeability” is used throughout the manuscript as a term describing the ability of the cuticle to allow water to diffuse through it. This term stands both for “cuticular permeance” measured with isolated cuticles and “minimum conductance” measured with whole leaves.

In a physical sense, permeability and permeance are not synonymous. The difference between the two terms is comparable to that of electrical conductivity (analogous to permeability) and conductance (analogous to permeance).

From an experiment measuring the diffusion of water across a cuticle as a function of time, the flux rate (in g m^-2^ s^-1^) can be obtained. This flux rate *J* is related to the water concentration difference *∆C* across the cuticle by

$$J=P\times\Delta C$$

*P* with dimensions of velocity (m s^-1^) is the permeance of the specific cuticle measured. It is a mass transfer coefficient and is the only descriptor for cuticular permeability because the thickness of the diffusion limiting barrier of the cuticle is unknown. This barrier is smaller than the total thickness of the cuticle. Permeance and minimum conductance are equivalent.

However, for homogeneous membranes of known thickness *x,* the permeability *p* with dimensions m^2^ s^-1^ can be obtained from

$$p=P\times x$$

The permeability *p* characterizes the property of a material to allow a substance to pass through it irrespective of the thickness of the membrane made from this material. For further discussion of this subject see Cussler 2009.

*Literature*

Cussler EL. 2009. Diffusion - Mass Transfer in Fluid Systems. Cambridge University Press, Cambridge.
