# Supplementary material for: Effectiveness of cuticular transpiration barriers in a desert plant at controlling water loss at high temperatures
Source: AoB Plants. 2016 May 6;8:plw027. doi: 10.1093/aobpla/plw027 (PMC4925923; doi:10.1093/aobpla/plw027)
Supplement: Supplementary Data [file supp_plw027_aobplants-15257-s_5.docx]

# File 2. Morphological leaf traits

### Material and methods

In the laboratory, the leaves were rehydrated in a humid chamber with the cut petioles immersed in water and the saturated leaf weights determined using an analytical balance (Sartorius MC-1 AC210S, Sartorius, Göttingen, Germany, precision 0.1 mg). The analysis of the kinetics of the saturation curves (Slavic 1974) revealed that leaves were fully saturated within 5 hours. The dry weight was determined after oven drying the leaves at 90° C for 24 hours. The actual fresh weights during leaf dehydration experiments were used to calculate the relative water content (RWC, dimensionless) and the relative water deficit (RWD, dimensionless) according to:

$RWD=1-RWC=1-\frac{actual fresh weight-dry weight}{saturated wieght-dry weight}$ (1)

For leaf area determination, leaves were scanned at high resolution (600 dpi) using a flatbed scanner connected to a computer. The projected leaf area was measured from the scanned leaf image using an image analysis software (Adobe Photoshop). Leaf area shrinkage (percentage loss of area, PLA) during leaf dehydration was measured for different dehydration levels:

$PLA=\left( 1-\frac{area at actual fresh weight}{area at saturated weight} \right)x 100$ (2)

Between the measurements, the leaves were allowed to desiccate on the bench. The maximum area shrinkage is given by the percentage decrease of the area in a dry leaf (Scoffoni *et al.* 2014). Stomata density was determined from leaf surface imprints using colourless nail polish (Sun *et al.* 2014). The imprints were observed under a microscope (Leica DMR, Leica Microsystems, Wetzlar, Germany) equipped with a digital camera (AxioCam MRc, Zeiss, Oberkochen, Germany). The number of stomata per area was counted from the digital images using an image analysis software (AxioVision, Zeiss).

### Results

Morphological leaf traits of *R. stricta* were determined to characterize its leaf properties in relation to adaptations to the unique habitat of this plant. The mean saturated weight of one leaf was 0.43 (± 0.15) g, the dry weight 0.11 (± 0.04) g and the projected (one-sided) leaf area 7.18 (± 2.23) x 10^-4^ m² (for all three values: n *=* 66). Stomata occurred on both leaf sides at a density of 121.95 (± 34.42) mm^-2^ on the adaxial and of 111.47 (± 33.49) mm^-2^ on the abaxial surface (n *=* 12).

For measuring water loss rates and minimum conductances, leaves were exposed to dry air that led to weight loss. Concomitantly, the area of the leaves shrank continuously (Fig. S1). The shrinkage curve has three distinct branches. In the relative water deficit (RWD) range from a fully saturated, turgid leaf to an RWD of 0.09 the leaf surface decreased by 1.8%. The percentage loss of leaf area (PLA) up to this RWD followed the regression equation: PLA = 20.138 (± 1.257) x RWD + 0.033 (± 0.045, ± SE of regression, F = 256.661, p < 0.001, r² = 0.928, n *=* 22 from 8 leaves). At higher RWD the leaf surface decreased by 4.8% down to a RWD of 0.60 according to the regression equation: PLA = 5.880 (± 0.525) x RWD + 1.273 (± 0.192, ± SE of regression, F = 125.380, p < 0.001, r² = 0.715, n *=* 52 from 15 leaves). At RWD > 0.60, PLA became progressively more pronounced. At full dehydration, the average maximum shrinkage of dry leaves reached 14.6% (± 2.1%, mean ± SD, n *=* 23).


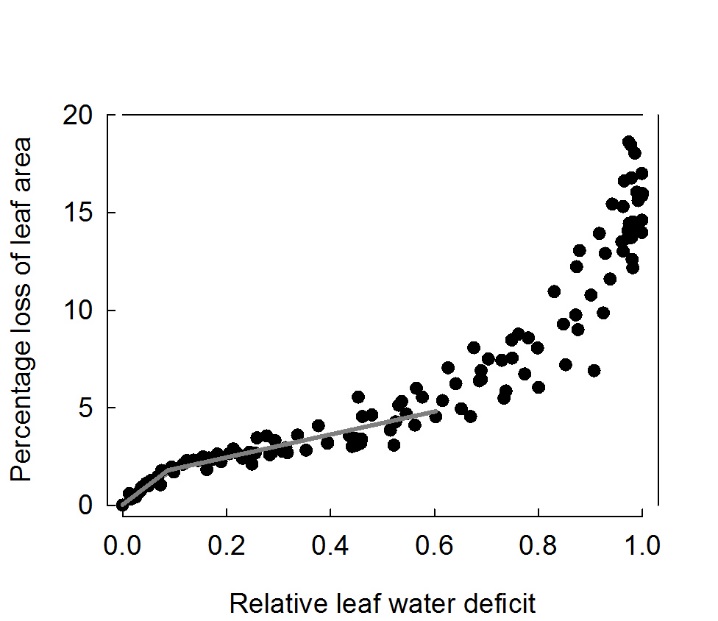


**Figure S1.** Percentage loss of leaf area as a function of relative water deficit (RWD). Each point represents an individual measurement obtained from dehydration experiments with a total of 16 leaves. Regression lines are fitted for RWD in the range between 0 and 0.09 as well as for RWD in the range between 0.09 and 0.60.

### Literature

Scoffoni C, Vuong C, Diep S, Cochard H, Sack L. 2014. Leaf shrinkage with dehydration: coordination with hydraulic vulnerability and drought. *Plant Physiology* 164:1772-1788.

Slavic B. 1974. Methods of studying plant water relations. Berlin: Springer.

Sun Y, Yan F, Cui X, Liu F. 2014. Plasticity in stomatal size and density of potato leaves under different irrigation and phosphorus regimes. *Journal of Plant Physiology* 171:1248-1255.
