# Supplementary material for: Effectiveness of cuticular transpiration barriers in a desert plant at controlling water loss at high temperatures
Source: AoB Plants. 2016 May 6;8:plw027. doi: 10.1093/aobpla/plw027 (PMC4925923; doi:10.1093/aobpla/plw027)
Supplement: Supplementary Data [file supp_plw027_aobplants-15257-s_6.docx]

# File 3. Leaf water potential and pressure-volume analysis

### Material and methods

A pressure chamber (PMS Instrument Company, Albany, Oregon, USA) was used to determine leaf water potential (Turner 1988). Saturated leaves were progressively dried on the bench. The water potential was repeatedly measured for different dehydration levels, and the corresponding actual fresh weights were determined immediately before and after water potential measurements. Water relation parameters were obtained from pressure-volume analyses (Bartlett *et al*. 2012, Sack *et al.* 2016). The leaf water potential (Ψ_leaf_ in MPa) represents the sum of the pressure potential (Ψ_P_) and the osmotic potential (Ψ_π_). For the construction of pressure-volume curves, the negative reciprocal value of Ψ_leaf_ is plotted against the RWD. The turgor loss point is the point on the graph where Ψ_P_ becomes zero; Ψ_π_ equals Ψ_leaf_ and Ψ_π_ is linearly related to RWD:

$-\frac{1}{\Psi_{\pi}}=-\frac{1}{\pi_{0}}+\frac{1}{\pi_{0} x (1-a_{f}}) x RWD$ (1)

The interception of this graph with the y-axis gives the osmotic potential at full saturation (π_0_). The symplastic water fraction is obtained from the interception with the x-axis, the apoplastic water fraction (a_f_) can be calculated (1 - symplastic water fraction). The pressure potential can be calculated as the difference between the measured water potential and the extrapolated osmotic potential. The plot of Ψ_P_ versus the relative symplastic water content (1 - a_f_) gives the modulus of elasticity (ε) as slope:

$\varepsilon=\frac{\Delta\Psi_{P}}{\Delta RWC} x (1-a_{f})$ (2)

### Results

The change of the leaf water potential during dehydration is an important trait for characterizing the reaction of a leaf to water loss. Plots of the leaf water potential (Ψ_leaf_) during dehydration allow to estimate critical water relation parameters of the leaf. When *R. stricta* leaves were exposed to dry air, Ψ_leaf_ declined rapidly at the beginning of the experiment and, starting at a RWD of approximately 0.09, at a lower rate. For estimating the water relation properties, pressure-volume curves were obtained by plotting the negative inverse Ψ_leaf_ versus RWD (Fig. S2). For the osmotic contribution to Ψ_leaf_, the regression equation Ψ_π_^-1^ = -0.878 (± 0.063) x RWD + 0.670 (± 0.016, ± SE of regression, F = 193.561, p < 0.001, r² = 0.785, n *=* 55 from 17 leaves) was established. The equivalent regression equation for the contribution of the hydrostatic pressure was Ψ_P_ = 14.968 (± 0.840) x (RWC) - 13.748 (± 0.800, ± SE of regression, F = 317.444, p < 0.001, r² = 0.906, n *=* 33 from 14 leaves) for the pressure term. Pressure-volume curves provide additional parameters which describe the water relation parameters of the leaf cells: (1) the relative water deficit at the turgor loss point (RWD_TLP_ = 0.09, dimensionless), (2) the osmotic potential at the turgor loss point (π_TLP_ = -1.69 MPa), (3) the osmotic potential at full saturation (π_0_ = -1.49 MPa), (4) the apoplastic water fraction (a_f_ = 0.24, Equation 1) and (5) the modulus of elasticity (ε = 11.42 MPa, Equation 2).


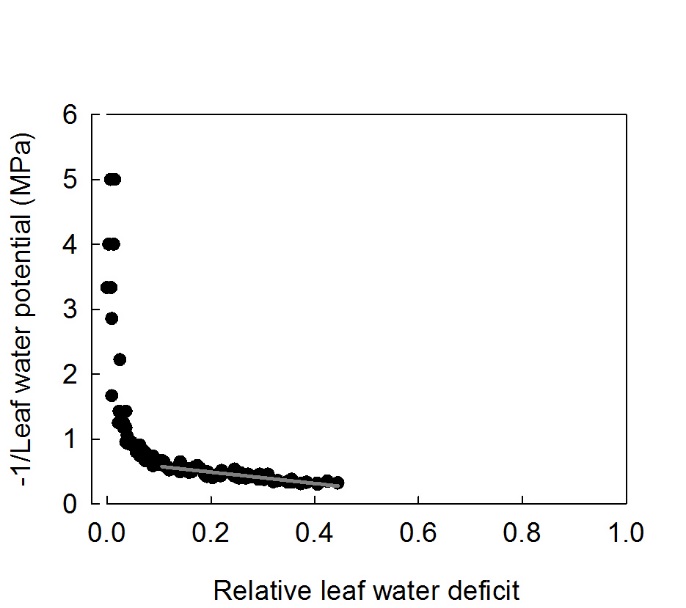


**Figure S2.** The pressure-volume curve of *Rhazya stricta* leaves. The negative reciprocal value of the leaf water potential (Ψ_leaf_) is plotted against the relative water deficit (RWD). Ψ_leaf_ is the sum of the osmotic potential (Ψ_π_) and the pressure potential (Ψ_P_). Each point represents an individual measurement obtained from dehydration experiments with a total of 18 leaves. The fitted line represents the results for the osmotic potential (Ψ_π_).

### Literature

Bartlett MK, Scoffoni C, Sack L. 2012. The determinants of leaf turgor loss point and prediction of drought tolerance of species and biomes: a global meta analysis. *Ecology Letters* 15:393-405.

Sack L, Pasquet-Kok J, PrometheusWiki contributors. 2016. Leaf pressure-volume curve parameters. PrometheusWiki, http://www.publish.csiro.au/prometheuswiki/tiki-pagehistory.php?page=Leaf pressure-volume curve parameters&preview=16 (accessed March 11, 2016).

Turner NC. 1988. Measurement of plant water status by the pressure chamber technique. *Irrigation Science* 9:289-308.
